# Stepwise construction of the path to doubled haploid breeding in sorghum

**DOI:** 10.1126/sciadv.aed5464

**Published:** 2026-05-22

**Authors:** Yi Sui, Yao Sun, Zixiang Cheng, Yali Li, Ke Li, Xiaohui Zhang, Tao Yin, Suhua Yang, Guiying Li, Minxuan Liu, Sanyuan Tang, Chuanyin Wu

**Affiliations:** ^1^ State Key Laboratory of Crop Gene Resources and Breeding, Institute of Crop Sciences, Chinese Academy of Agricultural Sciences, Beijing 100081, China.; ^2^ Institute of Wetland Agriculture and Ecology, Shandong Academy of Agricultural Sciences, Jinan 250100, China.; ^3^ Key Laboratory of Plant Molecular Physiology, Institute of Botany, Chinese Academy of Sciences, Beijing 100093, China.; ^4^ Institute of Genetics and Developmental Biology, Chinese Academy of Sciences, Beijing 100101, China.

## Abstract

Doubled haploid (DH) breeding greatly accelerates crop improvement, but efficient in vivo haploid induction has remained limited primarily to maize because readily available haploid inducers are lacking in other species. Here, we establish a complete DH breeding pipeline for sorghum by overcoming this key limitation. Using genome editing, we developed maternal haploid inducers that achieved haploid induction rates up to 16.04% in the P898012 background, with an average of 12.18% across 92 diverse crosses (15,156 seeds; various genetic backgrounds and environments). A dual-marker system combining fluorescence and pigmentation enabled reliable haploid identification. By optimizing a scalable emasculation protocol and using sorghum’s naturally high rate of chromosome doubling, we established an efficient in planta DH production process. This framework provides a practical, transferable approach to implementing DH technology in sorghum and other crops.

## Teaser

Gene-edited sorghum inducers enable efficient production of doubled haploid plants, advancing crop improvement.

## INTRODUCTION

Doubled haploid (DH) technology has transformed modern crop improvement by enabling the rapid generation of fully homozygous lines, eliminating the need for multiple generations of self-pollination (*1*). This acceleration shortens breeding cycles and facilitates efficient trait selection and variety development. Among available haploid production systems, including in vitro tissue culture, in planta haploid induction (HI) represents an alternative approach that is less genotype-dependent and operationally simpler in practice. However, widespread adoption of DH technology depends on achieving a practically applicable HI rate (HIR) that enables cost-effective and high-throughput production (*1*–*3*).

To date, maize remains the primary crop in which in planta HI has been widely adopted at a commercial scale, with the wide-hybridization (wheat pollinated by maize) system representing another major application (*4*). The discovery of the Stock 6 line, which exhibited a maternal HIR of ~1 to 2% (*5*), led to decades of breeding and genetic optimization, resulting in inducer lines with stable HIRs exceeding 10% (*6*). In parallel, integrated marker systems such as seed pigmentation and high oil content were developed for haploid identification, along with mechanical emasculation and chemical chromosome-doubling protocols (*7*, *8*). Together, these advances established a robust DH platform that underpins modern maize breeding and provided a foundation for applying similar systems to other crops.

High HIR in maize is controlled by multiple quantitative trait loci, with *qhir1* and *qhir8* being key contributors (*9*, *10*). The *qhir1* locus encodes a pollen-specific patatin-like phospholipase gene, *MATRILINEAL*/*PHOSPHOLIPASE A1/NOT LIKE DAD* (*MATL*/*ZmPLA1*/*NLD*), identified independently by three groups (*11*–*13*). This gene is conserved across monocots, and its knockout in other cereals yields ~3% HIR (*14*–*18*). The *qhir8* locus contains *ZmDMP*, a pollen-specific DUF679 domain membrane protein gene. Routine maize inducers harbor a single-nucleotide variant in *ZmDMP*, and double mutants of *MATL* and *ZmDMP* can reach ~6% HIR (*19*). While *ZmDMP* loss alone induces low HIR (0.1 to 0.3%) (*19*), its homologs in dicots also trigger HI, albeit at modest efficiencies that rarely exceed 3% (*20*–*33*). In dicots, which lack functional *MATL* orthologs, the *DMP* mutation alone is sufficient to induce haploids. In contrast, knockout of *OsDMP3* and *OsDMP6* in rice, alone, or combined with *osmatl* fails to confer noticeable HI or synergistic effect with *osmatl* (*34*). More recently, mutation of *PHOSPHOLIPASE D3* (*ZmPLD3*) in maize induced haploids at ~1% HIR and acted synergistically with *matl* and z*mdmp* (*35*). Despite these advances, maternal HIRs of ≥10%, necessary for practical DH breeding, remain confined to maize.

Sorghum (*Sorghum bicolor*) ranks as the world’s fifth most important cereal, cultivated across ~40 million ha in more than 100 countries. It is vital for food, feed, and biofuel production, particularly in arid and semiarid regions (*36*, *37*). Owing to its drought tolerance, low nutrient requirement, and minimal production costs, sorghum is a key crop for sustainable agriculture under climate stress (*38*–*40*). Accelerating its genetic improvement is therefore a global priority. Inspired by the maize Stock 6 model, a screen of 4000 sorghum accessions identified two accessions (SH001 and SH002) capable of maternal HI at 1 to 2% HIR (*41*), although the underlying gene(s) remain unknown. Despite sorghum’s close evolutionary relationship to maize, none of the known maize HI genes have been characterized or functionally validated in sorghum, leaving its potential for DH production unexplored.

Here, we establish a path toward commercial-scale DH breeding in sorghum. We generated single, double, and triple mutants of putative *MATL*, *DMP*, and *PLD3* homologs and systemically evaluated their HIR using engineered marker systems. Double mutants of *SbMATL* and *SbDMP* produced stable, breeding-level HIRs across diverse genetic backgrounds and environments. We further examined paternal and maternal effects on induction efficiency, identified a high frequency of spontaneous chromosome doubling in haploid plants, and optimized the emasculation protocol to enhance cross-pollination. Together, these advances establish a complete DH system in sorghum that, for many genotypes, operates without colchicine, positioning it as the next major crop suitable for large-scale DH breeding.

## RESULTS

### Fluorescent markers enable haploid embryo identification in sorghum

In maize, haploid embryo identification has relied on pigment-based markers (e.g., R1-nj and R1-SCM2) in commercial breeding programs because of their nontransgenic nature and recently fluorescent transgenic markers for earlier-stage detection in research settings (*42*–*49*). To establish a similar system in sorghum, we generated green fluorescent protein (GFP)–expressing lines in the P898012 and Tx430 backgrounds through *Agrobacterium*-mediated transformation with *CaMV35S-*driven GFP constructs. Hygromycin phosphotransferase and phosphinothricin acetyltransferase were used as selection markers for Tx430 and P898012, respectively (fig. S1A). Ninety-four independent transgenic events were obtained, and two stable lines, *P89GFP* and *TxGFP*, that exhibited consistent, heritable high-level GFP expression were chosen for subsequent experiments (fig. S1, B and C). Transferred DNA (T-DNA) insertions were mapped to chromosome 2 [75,331,531 base pairs (bp)] and chromosome 1 (4,617,987 bp) (fig. S1, D and E, and table S1).

Crosses between these GFP lines and the male-sterile line L407A demonstrated 100% GFP fluorescence transmission in hybrid seeds (fig. S2, A and B), and plants retained standard vigor and fertility (fig. S2, C and D). This system establishes a stable, reliable visual marker platform for identifying haploid sorghum embryos.

### Stacked mutations enable a scalable HI in sorghum

We identified *SbMATL* (*Sobic.001G348600*) as the sorghum’s ortholog of maize *MATL/ZmPLA*/*NLD* (92% amino acid identity). To determine whether *SbMATL* knockout induces haploidy as in maize, we constructed CRISPR-Cas9 vectors containing two single-guide RNAs targeting the second exon of *SbMATL* and transformed both *P89GFP* and *TxGFP* (fig. S3). Among 24 transformation events, 12 carried *SbMATL* edits of various types (table S2). Two frameshift mutants, *Sbmatl-1*;*TxGFP* and *Sbmatl-2*;*P89GFP*, were selected for evaluation.

**Fig. 1.**
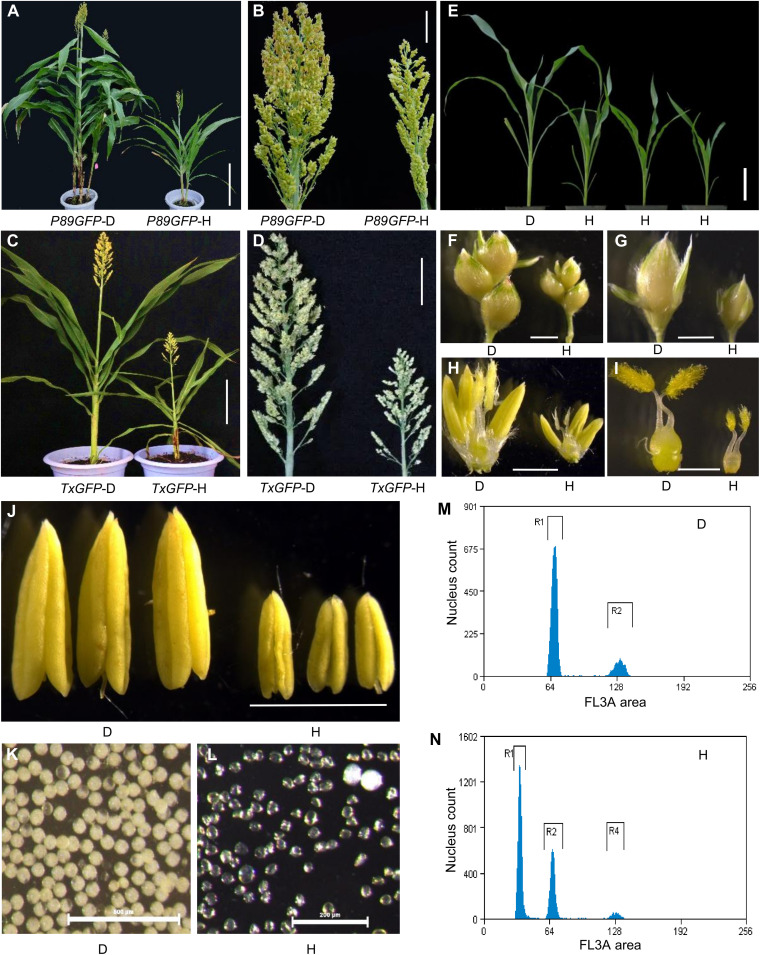
Morphological and cytological characteristics of haploid sorghum in two genetic backgrounds. (**A** to **D**) Representative haploid sorghum plant exhibiting reduced height [(A) and (C)] and smaller panicles [(B) and (D)] compared with diploid controls in the *P89GFP* [(A) and (B)] and *TxGFP* [(C) and (D)] backgrounds. (**E** to **L**) Morphological comparison of shoot (E), branches (F), spikelet (G), floret (H), pistil (I), anthers (J), and pollen grains [(K) and (L)] between haploid and diploid plants. (**M** and **N**) Flow cytometry analysis confirming ploidy status. Haploids (N) exhibit a fluorescence intensity peak approximately half that of diploids (M). The *x* axis represents the fluorescence signal (nuclear DNA content), and the *y* axis indicates the number of nuclei. Panels (D) and (H) denote diploid and haploid samples, respectively. Scale bars, 30 cm (A), 5 cm [(B) and (D)], 15 cm (C), 10 cm (E), 2 mm [(F) to (H)], 1 mm [(I) and (J)], 500 μm (K), and 200 μm (L).

From 142 selfed progeny of *Sbmatl-1*;*TxGFP* and 137 of *Sbmatl-2*;*P89GFP*, we identified 2 and 4 putative haploid plants, respectively. These plants were smaller than the wild type (WT) across multiple organs, displaying typical haploid morphology and pollen sterility (Fig. 1, A to L). Flow cytometry confirmed their haploid status (Fig. 1, M and N). No haploids were detected among 317 WT progeny (fig. S4).

**Fig. 2.**
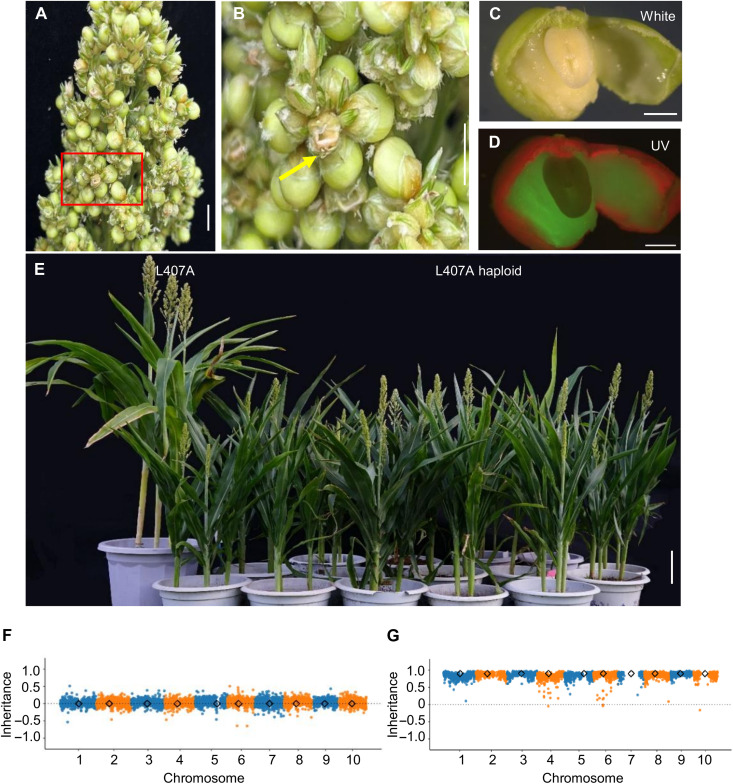
High-frequency and repeatable HI by cross-pollinated sorghum. (**A** and **B**) Representative phenotype of the male-sterile line L407A panicles pollinated by the *Sbmatl Sbdmp* inducer line (A) and enlarged red rectangle (B). Yellow arrows indicate aborted seeds. (**C** and **D**) Identification of putative haploid embryos using the GFP reporter: absence of GFP signal in embryos but strong GFP fluorescence in the surrounding endosperm under white (C) and UV (D) light. (**E**) Morphological comparison of a maternal haploid and its female parent (L407A). Haploids exhibit smaller vegetative and reproductive organs than diploids. (**F** and **G**) Representative genome dosage plots for L407A diploid (F) and haploid (G) plants from the cross L407A × *Sbmatl Sbdmp*; *P89GFP*. Data were calculated in 100-kb nonoverlapping bins across 10 chromosomes (Chr1 to Chr10). Each dot represents one bin; black diamonds mark centromere positions. Scale bars, 2 cm [(A) and (B)], 2 mm [(C) and (D)], and 10 cm (E).

Crosses of the male-sterile line L407A with *Sbmatl-1*;*TxGFP* or *Sbmatl*-*2*;*P89GFP* produced a notable proportion of aborted seeds (Fig. 2, A and B). The GFP marker enabled early identification of developing seeds containing putative haploid embryos—those lacking GFP in the embryos but retaining fluorescence in the endosperm (Fig. 2, C and D). Across five crosses, we dissected 3746 developing seeds and isolated 102 putative haploid embryos, corresponding to average HIRs of 1.83% (*Sbmatl-1*;*TxGFP*) and 5.66% (*Sbmatl-2*;*P89GFP*) (table S3). Some embryos were germinated on hormone-free Murashige and Skoog (MS) medium and grown to maturity (Fig. 2E and fig. S5), and flow cytometry confirmed their haploid status (fig. S6). These findings demonstrate that *SbMATL* knockout confers the haploid-induction ability but only at a basal level.

**Fig. 3.**
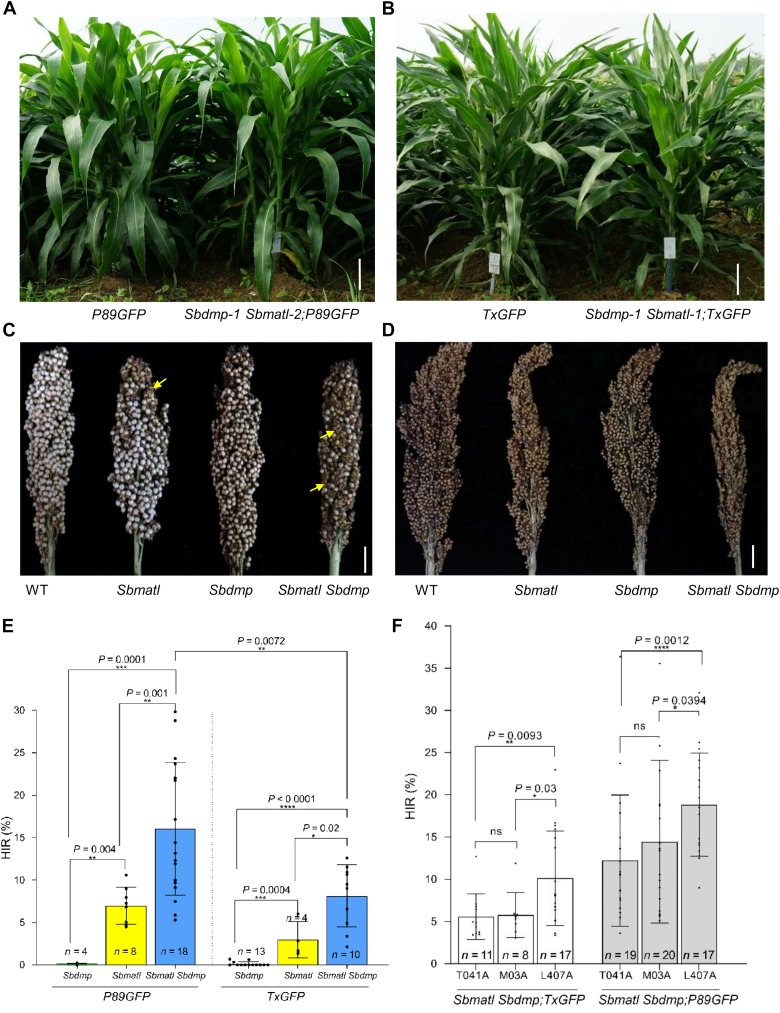
Mutation of *Sbmatl* and *Sbdmp* confers HI capability in two sorghum backgrounds. (**A** and **B**) Plant morphology comparison between *Sbmatl Sbdmp* inducer lines in *P89GFP* (A) and *TxGFP* (B) backgrounds. (**C** and **D**) Panicle phenotypes of WT, *Sbmatl*, *Sbdmp*, and *Sbmatl Sbdmp* double mutants in *P89GFP* (C) and *TxGFP* (D) backgrounds. Yellow arrows indicate aborted seeds. (**E**) HIR of L407A pollinated by *Sbdmp*, *Sbmatl*, and *Sbmatl Sbdmp* inducers in *P89GFP* and *TxGFP* backgrounds. The HIR of *Sbmatl Sbdmp* in P89GFP was higher than in *TxGFP* (table S11). Data are shown as the means ± SD; **P < 0.01, ***P < 0.001, and ****P < 0.0001 (two-sided Mann-Whitney test). (**F**) HIR of *Sbmatl Sbdmp* inducer lines (*TxGFP* and *P89GFP*) crossed with M03A, T041A, and L407A (table S12). Data are the means ± SD; ns, not significant; *P < 0.1, **P < 0.01, ***P < 0.001, and ****P < 0.0001 (two-sided Mann-Whitney test). Scale bars, 30 cm [(A) and (B)] and 2 cm [(C) and (D)].

In maize, *ZmDMP* mutation alone confers a negligible HIR, but in the *matl* background, it enhances HIR two- to threefold (*19*). Similarly, *DMP* homolog knockout plants in dicots yield ≤3% HIR (*20*–*33*). To examine this interaction in sorghum, we identified *SbDMP* (Sobic.*010G093200*), which shares 96% amino acid identity with *ZmDMP*, and created *Sbdmp* mutants in both the two GFP lines using CRISPR-Cas9 (fig. S7 and table S4). Crosses of L407A with these mutants yielded HIRs of 0.06% (*P89GFP*) and 0.13% (*TxGFP*), indicating a nearly negligible induction ability (Fig. 3F).

We next generated *Sbmatl Sbdmp* double mutants by knocking out *SbDMP* in the *Sbmatl-2*;*P89GFP* background (fig. S7 and table S4). Three double mutants—*Sbdmp-1 Sbmatl-2*;*P89GFP*, *Sbdmp-2 Sbmatl-2*;*P89GFP*, and *Sbdmp-3 Sbmatl-2*;*P89GFP*—were evaluated in crosses with L407A. From nine crosses (2028 developing seeds), we identified 320 haploid embryos, with HIRs ranging from 8.59 to 21.24% (16.26% on average) (table S5). Flow cytometry of randomly selected haploids confirmed their ploidy, distinguishing them from diploid hybrids (fig. S8). Morphological analysis revealed characteristic haploid phenotypes, inducing smaller stature and compact architecture (fig. S9). Genomic sequencing verified the maternal origin of the hybrid genomes by identifying only L407A-specific polymorphisms and the complete absence of P898012-specific polymorphisms (Fig. 2, F and G).

We also investigated *SbPLD3* (*Sobic.009G062600*), the sorghum homolog of maize *ZmPLD3*, which enhances HI in *matl zmdmp* backgrounds. Unlike maize, we successfully obtained homozygous *Sbpld3* mutants in *Sbdmp-1 Sbmatl-2*;*P89GFP* backgrounds (fig. S10A and table S6), but mutation of *SbPLD3* did not improve HI in either *Sbmatl Sbpld3* double or *Sbmatl Sbdmp Sbpld3* triple mutants (fig. S10, B and C, and table S7), indicating that *PLD3*-mediated synergy is not conserved in sorghum.

The *Sbmatl Sbdmp* double mutants displayed normal vegetative morphology (Fig. 3, A and B), with pollens containing normal starch accumulation as indicated by I_2_/KI staining (fig. S11, A and D). However, pollen viability and germination ability assays indicated 13.18 and 12.73% reduction, respectively, in the double mutants compared to the WT control (fig. S11, B, C, E, and F). In addition, the pollen competition assay showed that the *Sbmatl Sbdmp* pollens were less competitive, contributing to 28.50 to 45.40% seed sets in four panicles pollinated (fig. S12 and table S8). Seed set rates in *Sbmatl Sbdmp* mutants averaged 24.62% (range: 10.98 to 38.89%), lower than those in *Sbmatl* (52.07%), *Sbdmp* (64.44%), or WT plants (98.81%) (Fig. 3, C and D, and table S9). It is noteworthy that *Sbmatl Sbdmp* lines were produced through three stepwise cycles of in vitro culture (including introduction of the transgene marker and editing of *SbMATL* and *SbDMP*), which might introduce somatic variations related to pollen reproductivity.

Maternal panicles pollinated by inducer lines exhibited high rates of abnormal seed development, most severe in crosses using *Sbmatl Sbdmp* inducers (fig. S13). Abnormalities included pearl-like embryos arrested at early stages (fig. S14), embryo-less seeds (fig. S15, A to D), malformed embryos (fig. S15, E to H), and malformed endosperms (fig. S15, I to P). GFP-positive tiny embryos lacking endosperm likely resulted from single fertilization of the egg cell (fig. S14, C and D). In contrast, GFP-negative embryos were often haploid, suggesting that HI occurs soon after pollination (fig. S14, E and F).

Whole-genome sequencing of these embryos revealed paternal chromosome loss and frequently biased paternal genome allocation (figs. S16 and S17). Among 23 GFP-negative putative haploids (14 morphologically normal and 9 defective), all 14 normal embryos were confirmed as true haploids. Of the nine abnormal embryos, six showed maternal genome origin, whereas three showed biparental genome origin but lacking paternal chromosome 2 (fig. S18). The inducer chromosome 2 carries the *GFP* transgene insertion (fig. S1E). This finding provides direct evidence that embryo abortion is associated with inducer chromosome loss during HI. A higher HIR was usually correlated with a higher seed abortion frequency (Fig. 3, C to F; fig. S19; and table S9).

To compare HIR at early versus mature seed stages, we divided *Sbmatl* single inducer–pollinated L407A panicles into two halves, harvesting one at 12 to 25 days after pollination and the other at maturity (>40 days) (fig. S20A). Haploids were identified by the absence of GFP signal in developing embryos or in germinating shoots (fig. S20, B to D). HIR measured during early seed development was slightly higher than that measured during seed germination (table S10), suggesting that a subset of early haploid embryos aborts during later seed development.

### HI efficiency varies between sorghum genotypes

To assess genotypical variation in HIR, we generated additional *Sbmatl Sbdmp* double mutants by knocking out *SbDMP* in the *Sbmatl-1*;*TxGFP* background. The resulting *Sbdmp Sbmatl-1*;*TxGFP* lines were compared with *Sbdmp Sbmatl-2 P89GFP* lines. Of 2262 developing seeds from 10 crosses, we identified 188 putative haploid embryos, corresponding to HIRs ranging from 3.37 to 12.61% (mean, 8.80%) in the *TxGFP* background (table S11).

Overall, the average HIR was 8.08% for the Tx430 background and 16.04% for the P898012 background based on the analysis of 4147 developing seeds from 27 crosses (Fig. 3E and table S11). As observed previously, stacked mutations in Tx430 also led to reduced seed set and developmental abnormalities (Fig. 3D and figs. S13 and S19).

We next examined HIR variation between maternal germplasms. Two additional male-sterile lines, M03A and T041A, were crossed with *Sbmatl Sbdmp* lines in both *P89GFP* and *TxGFP* backgrounds. Analysis of 15,156 seeds from 92 crosses yielded average HIRs of 10.13%/18.84% (L407A), 5.76%/14.45% (M03A), and 5.57%/12.20% (T041A) in the *TxGFP*/*P89GFP* backgrounds, respectively (Fig. 3F, fig. S21, and table S12). These results indicate that the maternal effect on HIR also exists, which varies among different genotypes in sorghum.

### Environmental impact on HI in sorghum

Initial HI experiments were conducted under potted conditions in Beijing (116°20′E, 39°56′N). To evaluate environmental influences, we systematically analyzed HIR of *Sbmatl Sbdmp* double mutants across multiple locations and years (2021 to 2025). Across 184 crosses and 37,488 germinated seeds, 5854 haploids were identified (table S13).

**Fig. 4.**
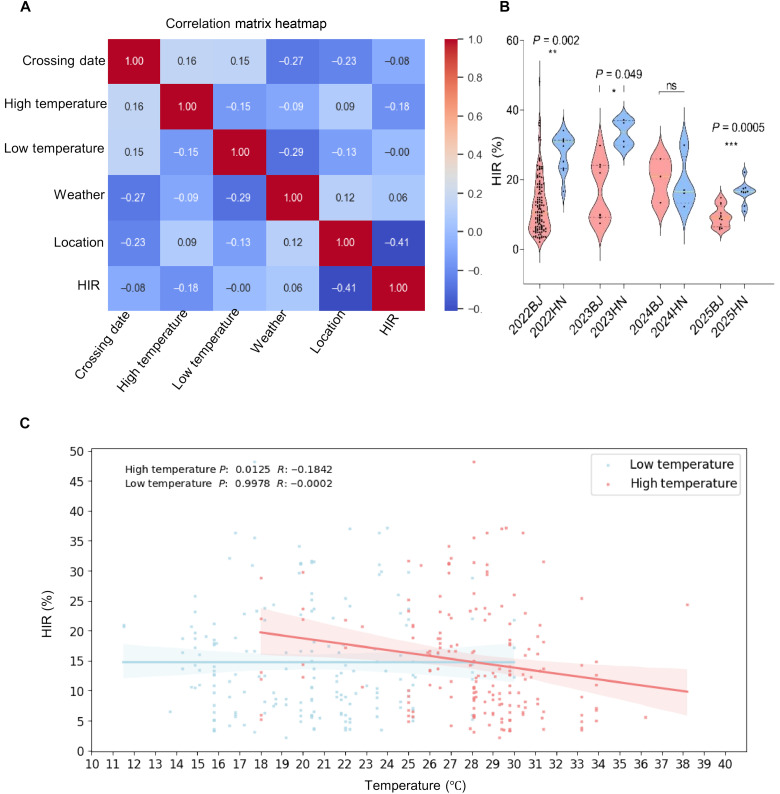
Environmental correlates of HIR in sorghum. (**A**) Spearman correlation matrix of meteorological and geographical variables. HIR shows a moderate positive correlation with location (ρ = 0.41) and a weak negative correlation with high temperature (ρ = −0.18). (**B**) Comparative HIR analysis between Beijing and Hainan across four growing seasons (2022 to 2025). Data are shown as the means ± SEM (***P < 0.001, two-tailed *t* test). (**C**) Linear regression analysis confirmed a significant negative correlation between high temperature and HIR (*P* < 0.05), while low temperature had no significant effect (*P* > 0.05). *R* denotes the correlation coefficient. Data were obtained from multienvironment trials conducted in Beijing and Hainan from 2021 to 2025 (table S13).

Geographical location was the strongest determinant of induction efficiency (Fig. 4A). Field trials in Hainan (109°17′E, 18°36′N) produced significantly higher HIRs than those in Beijing in three of four tested years (*P* < 0.05) (Fig. 4B). Correlation analysis confirmed that geographic location positively correlated with HIR (ρ = 0.41) (Fig. 4A).

Temperature was also a key environmental factor. Regression analysis revealed a significant negative correlation between elevated temperatures and HIR (*P* < 0.05), with optimal induction occurring at moderate temperatures (Fig. 4C). Low temperatures did not significantly affect HIR (*P* > 0.05). Combined environmental and geographical analyses showed that HIRs were the highest during Hainan’s winter when moderate temperatures and favorable field conditions coincided. These results indicate that HI efficiency in sorghum is shaped by interactions between genotype (*Sbmatl Sbdmp*) and environmental variables, particularly temperature and geographical location.

### Chemical emasculation enables controlled cross-pollination for HI in sorghum

Because sorghum is a self-pollinated hermaphroditic species, successful HI requires reliable emasculation of female parents to ensure controlled cross-pollination with inducer lines. We first tested a humidity-based emasculation method, in which panicles were enclosed in plastic bags and sprayed with water before anthesis (*50*). This method failed to prevent self-pollination effectively (table S14). Manual emasculation by physical anther removal also resulted in frequent self-fertilization, confirmed by progeny fluorescence assays using *P89GFP* pollen donors (table S15).

We then evaluated the pollen development inhibitor trifluoromethanesulfonamide (TFMSA) as a chemical emasculant (*51*). TFMSA application at varying concentrations, with or without surfactants (Tween 20 and/or Silwet L-77), induced complete male sterility in some treated plants, with optimal application time being 35 to 46 days before flowering (table S16 and fig. S22, A to E).

Pollination with GFP-expressing pollens on TFMSA-treated plants yielded 2445 seeds, >95% of which displayed GFP signals, further confirming the effectiveness of the chemical emasculation and successful cross-pollination (table S17). A repeated application with an interval of 20 days resulted in increased male emasculation rates, thereby effectively accommodating variation in flowering time (table S18). Subsequently, 36 sorghum varieties and 10 F_1_ hybrids (65 crosses total) were chemically emasculated and pollinated with *Sbmatl Sbdmp* inducers, producing 441 haploid candidates from 4343 seeds (average HIR = 12.37%) (table S19).

Field trials using six elite F_1_ breeding lines as female parents further validated this approach. TFMSA-treated plants pollinated with *Sbmatl Sbdmp* inducers yielded 2979 hybrid seeds, from which 276 haploids were identified on the basis of GFP screening (fig. S22F). Flow cytometry confirmed the haploid status of randomly selected plants. Collectively, these experiments confirm TFMSA-mediated chemical emasculation as an effective, reproducible, and scalable method for controlled cross-pollination in sorghum, enabling efficient in planta HI and DH breeding.

### Development of a visual marker system for haploid identification in sorghum

During initial screening of progeny from crosses between L407A and *Sbdmp-1 Sbmatl-2*;*P89GFP* lines, we observed variation in shoot base pigmentation at the germination stage. Most seedlings displayed pigmentation resembling that of the inducer parent, while a small subset exhibited light pigmentation similar to L407A, enabling preliminary visual identification of putative haploids (fig. S23). However, this phenotypic distinction was inconsistent across different genetic backgrounds. Moreover, reliance on GFP fluorescence required ultraviolet (UV) dissection microscopy, limiting its use in routine breeding programs.

**Fig. 5.**
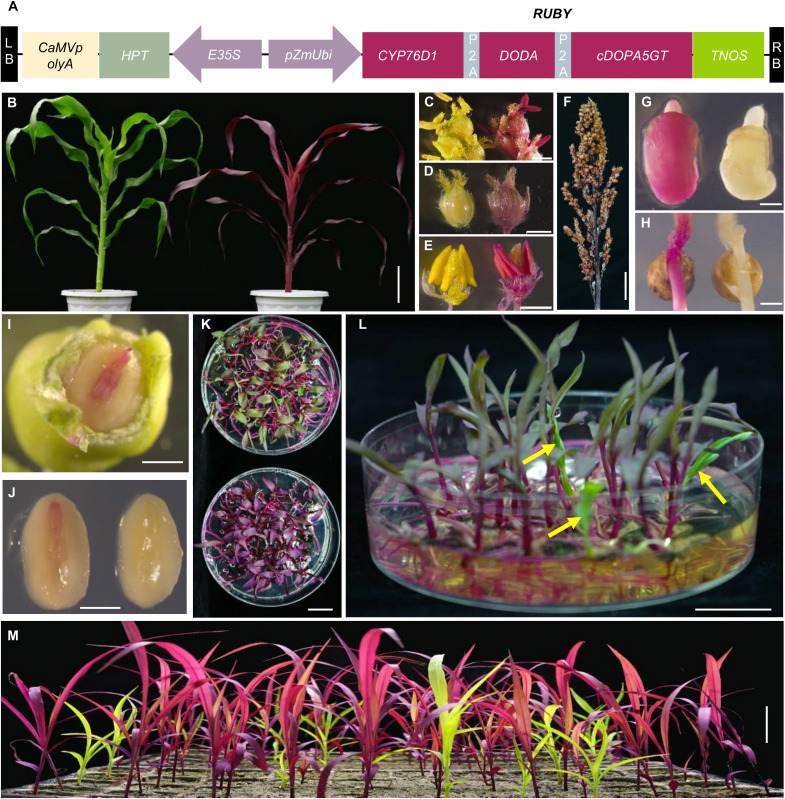
The dual-marker system enables visual haploid identification in sorghum using RUBY. (**A**) Schematic of the RUBY expression construct integrated into the sorghum inducer line. (**B** to **E**) Visible pigmentation differences between *Sbdmp-1 Sbmatl-2*;*P89GFP* (left) and *Sbdmp-1 Sbmatl-2*;*P89GFP-RUBY* (right) inducer lines in a whole plant (B), panicles (C), floret (D), and anthers (E). (**F**) Mature panicle of *Sbdmp-1 Sbmatl-2*;*P89GFP-RUBY* showing strong red pigmentation. (**G** and **H**) Embryos (G) and germinating seeds (H) from crosses with RUBY (right) and non-RUBY (left) inducers. (**I**) Embryo from L407A × *Sbdmp-1 Sbmatl-2*;*P89GFP-RUBY* cross showing RUBY pigmentation under white light. (**J**) Dissected diploid embryo tip displaying ruby color (left) versus putative haploid lacking pigmentation (right). (**K**) Visual identification system showing haploid (green) and diploid (RUBY) seedlings from cross-pollination (top) versus self-progeny (bottom). (**L**) Magnified view of haploids (yellow arrows) lacking RUBY pigmentation. (**M**) Germination of hybrid seeds from L407A × *Sbdmp-1 Sbmatl-2*;*P89GFP-RUBY* in soil. Scale bars, 10 cm (B), 2 mm [(C) to (E) and (G)], 1 mm [(F), (H), and (I)], 2 cm [(J) and (K)], and 5 cm (L).

To overcome these constraints, we introduced an engineered pigmentation cassette, RUBY, which induces betalain accumulation and vivid RUBY coloration (*52*), together with an NPTII selectable marker into the *Sbdmp-1 Sbmatl-2*;*P89GFP* line lacking *NPTII* (Fig. 5A). The resulting dual-marker inducers (*Sbdmp-1 Sbmatl-2*;*P89GFP-RUBY*) exhibited strong red pigmentation across tissues, including embryos and germinating seeds (Fig. 5, B to H).

Identification of haploid embryos or germinating haploid shoots was easily achieved (Fig. 5, I to M, and figs. S24 and S25). Pollination of L407A with these double-marker lines yielded 84 haploid seedlings from five crosses, corresponding to an average HIR of 20.08% (Table 1). This system enables reliable, equipment-free identification of haploids at early developmental stages. In addition, simplified *Sbdmp-1 Sbmatl-2*;*P89-RUBY* inducer lines were created by segregating out the GFP transgene through backcrossing with WT plants, providing a versatile, field-ready tool for DH sorghum breeding.

**Table 1.** HIRs confirmed in crosses between L407A and the *sbdmp-1 sbmatl-2*;*P89GFP-RUBY* inducer line.

No. of crossings	Crossing date	Total germinated diploid seeds	Total germinated haploid seeds	Total ungerminated aborted seeds	Total germinated seeds	Total seeds	HIR (%)
1	2 July 2023	101	25	37	126	163	19.84
2	31 August 2023	71	19	18	90	108	21.11
3	31 August 2023	42	15	9	57	66	26.32
4	11 September 2023	83	16	7	99	106	16.16
5	15 September 2023	44	9	21	53	74	16.98
Average							20.08

### Spontaneous chromosomal doubling enables efficient DH production in sorghum

Chromosome doubling in haploids is crucial to produce DH seeds for subsequent trait evaluation. In haploid maize, spontaneous chromosome doubling occurs at low frequency, and therefore, chemicals, such as colchicine and herbicides, are often used for large-scale production of DH plants. The established efficient HI system enabled us to extensively examine spontaneous chromosome doubling in sorghum haploids.

To assess female fertility in haploids, we conducted controlled pollinations of 40 L407A haploids with WT pollen. Thirty-seven plants produced seeds, ranging from 1 to 41 per plant (table S20), indicating substantial but variable female haploid fertility. To test autonomous doubling, 93 bagged haploid panicles from nine genetic backgrounds were evaluated under selfing conditions. Fifty-nine panicles (63.44%) set seeds, with yields ranging from 1 to 187 seeds per panicle (table S21), confirming widespread spontaneous doubling.

**Fig. 6.**
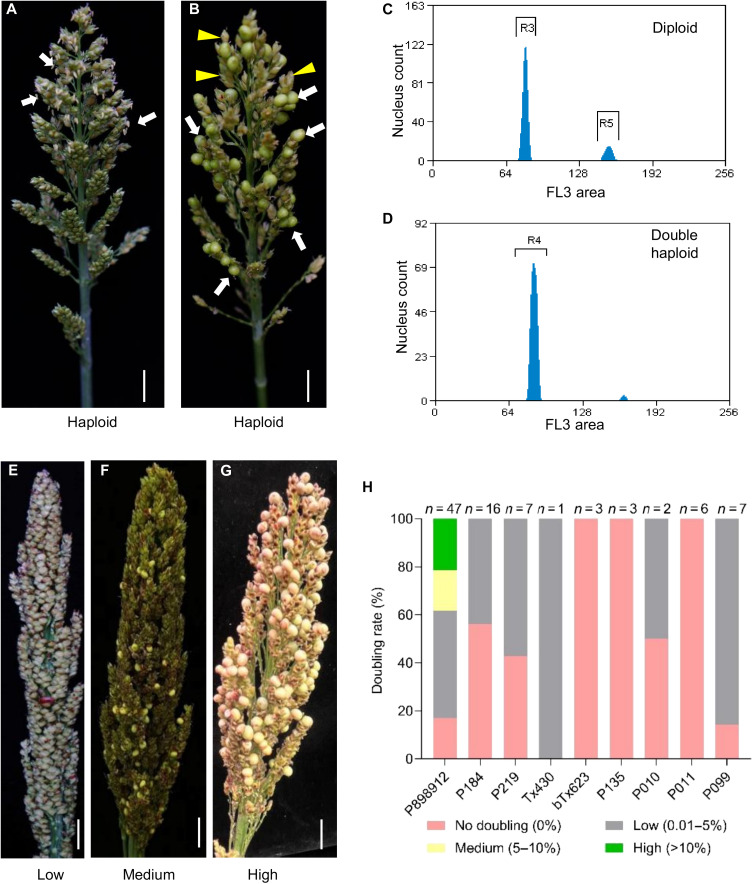
Spontaneous chromosome doubling enables direct DH production in sorghum. (**A** and **B**) Spontaneous chromosome doubling restores fertility in a haploid P898012 plant, as shown by viable pollen [(A), white arrows] and fertile diploid spikelets [(B), white arrows] among sterile haploid spikelets [(B), yellow arrowheads]. (**C** and **D**) Flow-cytometric confirmation of ploidy levels in a diploid control (C) and a spontaneously DH plant (D). The *x* axis indicates the nuclear fluorescence peak, and the *y* axis represents the number of counted nuclei. (**E** to **G**) Representative panicles categorized by doubling efficiency: (E) low (0.01 to 5% seed set), (F) medium (5 to 10%), and (G) high (>10%). (**H**) Distribution of spontaneous doubling efficiency across nine genetic backgrounds. Plants were classified into the four categories including three in (E) to (G); *n* indicates the total plants per genotype. Scale bars, 0.5 cm [(A), (B), and (E) to (G)].

This phenomenon was strongly genotype-dependent. Among the nine genotypes tested, P898012 displayed the highest fertility, characterized by visible pollen shedding (Fig. 6A) and abundant seed set (Fig. 6B). Flow cytometry confirmed somatic genome doubling in these fertile haploids (Fig. 6, C and D). Haploids were classified into four categories on the basis of their seed set: none (0%), low (0.01 to 5%), medium (5 to 10%), and high (>10%) (Fig. 6, E to G). Distribution analysis revealed that BTx623, P135, and P011 showed no doubling; Tx430 and P099 exhibited low rates; P184, P219, and P010 displayed mixed none-to-low rates; and only P898012 produced individuals in the high (>10%) class (Fig. 6H).

To test applicability in breeding materials, we analyzed haploids from two F_1_ hybrids. All ZH0639 × ZH0612 haploids produced seeds, with one plant setting 119 seeds (3.5% seed set rate; table S22). In contrast, only half of the P184 × Tx430 haploids doubled, with lower efficiency. These results confirm that spontaneous chromosome doubling operates in elite germplasm, albeit with strong genotype dependence.

**Fig. 7.**
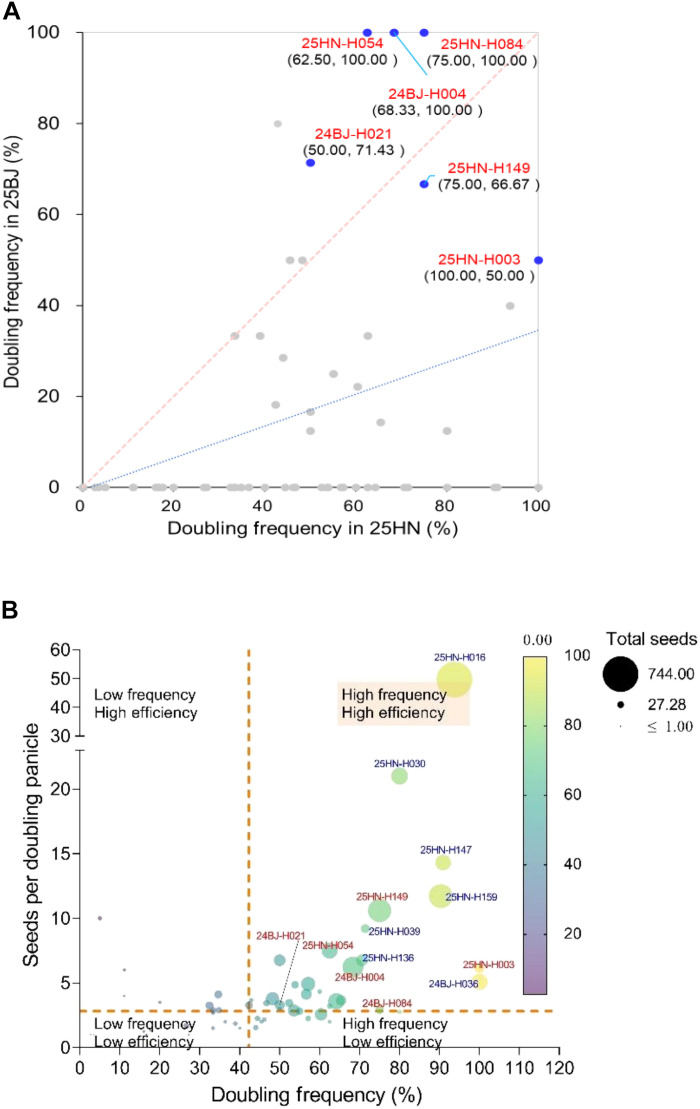
Identification of elite sorghum haploid genotypes based on spontaneous-doubling stability and efficiency. (**A**) Stability of spontaneous-doubling frequency across two environments (25HN and 25BJ) in 74 haploid genotypes. The dashed line (*y* = *x*) represents perfect stability. Six genotypes (labeled) maintained >50% doubling efficiency in both environments. (**B**) Two-dimensional profiling of doubling performance in the 25HN environment, integrating doubling frequency (*x* axis) and reproductive efficiency (*y* axis). The bubble size represents the total seed output. Dashed lines mark median values. Genotypes located in the upper-right quadrant exhibit high-frequency, high-efficiency performance. Color coding indicates selection overlap: red, elite in both panels; blue, elite only in (B).

For breeding applications, the consistency of spontaneous chromosome doubling in haploids across geographic regions is essential. We therefore evaluated 73 haploid plants isolated from an F_2_ population from the cross between *Sbdmp Sbmatl-1*;*TxGFP* and *Sbdmp Sbmatl-2*;*P89GFP* lines across two environments, 2024–2025 winter in Hainan, South China, and 2025 summer in Beijing (25HN and 25BJ). Doubling efficiency in a given haploid was assessed on the basis of panicles with seed set over total panicles examined for that plant. Six genotypes maintained doubling efficiency above 50% in both environments, and three achieved complete (100%) doubling in 25BJ while maintaining high rates in 25HN (Fig. 7A and table S23). Two-dimensional profiling of doubling frequency and reproductive output identified ideal genotypes combining stability and productivity (Fig. 7B and table S23).

To leverage spontaneous doubling for DH production, we tested two complementary strategies. For low-efficiency genotypes, field transplantation combined with targeted tiller management increased the panicle number and overall seed yield (fig. S26). In addition, we recently treated 15-day-old haploid embryos with 0.005 and 0.01% colchicine, respectively. The preliminary evaluation on seedlings (17 days after germination) by flow cytometry suggests the substantial effectiveness of the colchicine treatment on chromosome doubling. Compared to the untreated group, where the haploid density peak was dominant, the diploid intensity peak became dominant in the two treated groups, with the 0.01% colchicine working better (figs. S27 and S28). Those plants are being maintained for seed setting to confirm this observation. Together, these two strategies can be useful to produce DH seeds in low-fertility haploid plants.

We grew 425 seeds from 40 F_1_-derived haploids to maturity and observed fully fertile DH plants (fig. S29). These results confirm that sorghum’s intrinsic capacity for spontaneous chromosome doubling provides a robust and scalable mechanism for DH production.

**Fig. 8.**
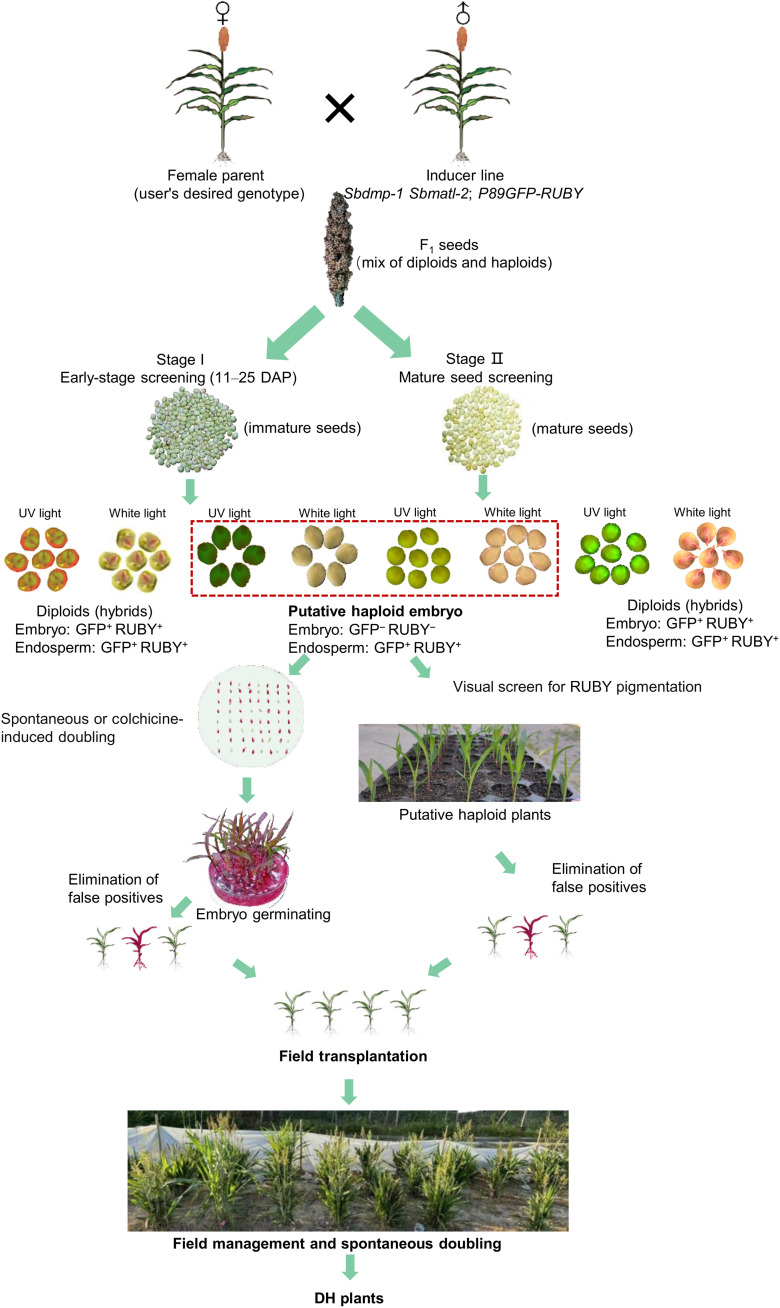
Workflow for a dual-visual marker pipeline for haploid identification and DH production in sorghum. Pollination with the inducer line *sbdmp-1 sbmatl-2*;*P89GFP-RUBY* enables a two-stage screening strategy for haploid identification and doubled-haploid production. Haploid embryos are distinguished from diploid hybrids by the absence of both GFP fluorescence and RUBY pigmentation, while the surrounding endosperm retains marker expression. Early-stage screening [11 to 25 days after pollination (DAP)]: Putative haploid embryos (lacking GFP/RUBY signals) are visually identified under UV or white light and directly transferred to culture medium—with or without colchicine—for chromosome doubling and plant germination. Mature-seed screening: Germinating seeds are examined for the absence of RUBY pigmentation in the embryo and for color in the endosperm. Putative haploids are transplanted to the field, where the persistent RUBY marker allows ongoing ploidy verification and removal of false positives during growth.

Collectively, this study establishes a complete, in planta DH breeding system for sorghum. By integrating efficient haploid inducers, practical visual markers, and spontaneous doubling strategies—both in the field and in vitro—we provide a reliable and genotype-adaptive framework for DH breeding. This system (Fig. 8) closes the final technological gap in sorghum DH production and offers a transferable model for developing similar pipelines in other major cereals.

## DISCUSSION

DH breeding represents a major advance in modern crop improvement, enabling rapid development of fully homozygous lines (*53*). However, establishing a functional DH system for sorghum has long been hindered by four interconnected barriers: low HIRs, inefficient haploid identification, labor-intensive emasculation, and dependence on chemical chromosome doubling (*41*, *54*). In this study, we systematically resolved each of these constraints through a stepwise engineering approach. By applying CRISPR-Cas9 to generate high-efficiency inducer lines with average HIRs exceeding 15%, we established a foundation for practical implementation. We further characterized the genetic and environmental factors influencing induction efficiency, integrated dual visual markers for reliable haploid identification, introduced a scalable chemical emasculation method, and used sorghum’s naturally high rate of spontaneous chromosome doubling. Together, these advances constitute a complete in planta DH pipeline that achieves efficiencies comparable to maize while maintaining practical applicability in breeding settings. Although further refinement remains possible, this work transforms DH breeding sorghum from a conceptual challenge into an operational system and offers a transferable framework for other crops.

The engineered inducer lines and supporting data knowledge generated here provide both a practical resource for sorghum breeders and a template for developing regionally adapted inducers. Maize DH breeding, refined over decades since the discovery of Stock 6, demonstrates the potential of this technology. Our results show that such progress can now be condensed into a few years through targeted editing of key haploid-induction genes. Future efforts can further accelerate optimization using multiplexed CRISPR systems (*55*) and transformation booster genes (*56*), allowing rapid adaptation of induction lines across diverse germplasms.

Our genetic analysis identified the principal components of the HI pathway in sorghum, revealing both conserved and species-specific features. Mutation of *SbMATL* alone induced haploid formation, confirming its conserved function as a central regulator in cereals, including maize (*11*–*13*), foxtail millet (*15*), wheat (*14*, *17*), sugarcane (*18*), and rice (*16*). We further demonstrated that *SbDMP* acts synergistically with *SbMATL*, raising HIRs above 12.18%, a threshold suitable for commercial breeding. While MATL is absent in dicots and DMP orthologs show limited activity in rice (*34*, *57*), our data suggest that the MATL-DMP module constitutes a conserved core of the HI mechanism within the sorghum-maize lineage. In contrast, *SbPLD3* did not enhance HIR when mutated in the *Sbmatl Sbdmp* background, indicating that not all components are conserved across species. Other candidate genes identified in related systems, such as *ZmPOD65* (*58*), Zm*Gex1* (*59*), *HAP*-*LESS2* (*HAP2*)/*GENERATIVECELLSPECIFIC1* (*GCS1*) (*60*, *61*), *KOKOPELLI* (*KPL*) (*62*), *GYNOECIUM-EXPRESSED PHOSPHOLIPASE AII* (*pPLAII*) (*63*), *OsMATL2* (*64*), *AtECS1*/*AtECS2* (*65*, *66*), *PARTHENOGENESIS* (*PAR*) (*67*), and *BBM* (*68*–*70*), represent promising targets for future functional screening in sorghum, particularly for identifying maternal-effect contributors to induction. This comparative framework establishes sorghum as a model system for finding new mechanisms beyond those characterized in maize.

The mechanistic basis of HI, specifically the fate of the paternal genome following fertilization, remains unsolved. In the *Sbmatl Sbdmp* double mutant, we observed reduced seed set and increased embryo-less seeds concurrent with higher HIR, suggesting that fertilization and genome stability are clearly linked. Double mutants produced seed set rates of 24.62% (P898012) and 26.99% (Tx430), markedly lower than *Sbmatl* single mutants (52.07% in P898012 and 70.65% in Tx430) (Fig. 3, C and D, and table S9). This reduction correlated with a >30% frequency of embryo-less seeds (fig. S12). Two complementary mechanisms likely underlie these outcomes: (i) chromosome fragmentation after fertilization and (ii) failed fertilization events (*1*, *3*). Whole-genome sequencing of GFP-negative embryos revealed partial paternal chromosomes (fig. S17), supporting the first mechanism. Meanwhile, reduced pollen viability in double mutants (fig. S11) indicates incomplete gamete fusion as a second possible contributor. The combined evidence suggests that haploid formation in sorghum involves both pre- and postfertilization disruptions—impaired pollen function and parental genome elimination—producing outcomes that range from embryo-less seeds to viable haploids. Similar patterns in other species, including mutations in *GEX1* (*59*) and *HAP2* (*61*), reinforce that HI often arises from perturbations in fertilization and early zygotic development.

Our dual-marker (GFP/RUBY) system provided high-resolution visualization of early embryogenesis, revealing important technical insight: Not all GFP-negative embryos are true haploids. Some diploid abortive embryos displayed segmental deletions of parental chromosomes, mimicking haploidy. This observation emphasizes the need for genomic or flow-cytometric validation to ensure ploidy accuracy. It also broadens the mechanistic understanding of HI as part of a wider “genome instability syndrome,” encompassing whole-genome elimination, segmental loss, and defective endosperm development triggered by inducer pollen.

The stability of HI depends on both genetic and environmental interactions. We identified genotype-dependent variation in HIR and demonstrated that induction efficiency reflects a complex interplay between paternal genotype, maternal background, and environment. The P898012 genotype exhibited particularly favorable characteristics—high induction receptivity and strong spontaneous chromosome doubling—making it an ideal foundation for future breeding. Environmental factors, notably geography and temperature, further modulated efficiency: HIRs peaked under moderate conditions in Hainan and declined under high-temperature stress. Recognizing these relationships allows the rational design of geographically optimized induction systems and environmental management strategies.

Although the use of transgenic markers can facilitate efficient identification of haploids as demonstrated in this study, such an approach may trigger biosafety regulation and necessitate stewardship and compliance protocols. In this case, the RUBY marker is preferred because a RUBY plant can be easily seen in field if the transgene is spread to other germplasms (fig. S30). For a non–genetically modified organism approach, markers can be developed on the basis of the genomic sequence of inducer and breeding germplasms, and haploids can be screened out by genotyping with the molecular markers. Alternatively, pigment markers may be exploited, as done in maize. Actually, a candidate gene responsible for purple leaf sheath and its associated molecular marker have been reported in sorghum (*71*), which holds promise to introduce pigment into inducer lines with marker-assisted selection.

Last, sorghum’s unique biological characteristics can be used to maximize DH production. Its high tillering capacity and continuous panicle formation enable flexible scheduling of emasculation and pollination, minimizing environmental constraints such as temperature spikes. Controlled environments or tropical regions can support year-round production. For genotypes with limited spontaneous doubling, haploid embryos can be germinated on colchicine-containing medium as a supplementary approach (figs. S27 and S28). Together, these strategies provide a practical roadmap for commercial DH deployment in sorghum.

## MATERIALS AND METHODS

### Plant material

Sorghum (*S. bicolor*) inbred lines Tx430 and P898012 were used for all transformation experiments. Plants were grown in 30- by 30-cm pots under natural summer conditions in Beijing (116°20′E, 39°56′N) or in a greenhouse during winter with temperature maintained at 28° to 34°C (day) and 20° to 24°C (night). The photoperiod was set to 14-hour light/10-hour dark. Male-sterile lines L407A, M03A, and T041A, along with fertile inbred lines (table S24), were cultivated either in pots or in field plots under natural summer conditions in Beijing and under field conditions in Yazhou, Hainan Island (109°17′E, 18°36′N) during winter. All plants were managed using standard agronomic practices.

### Vector construction

Candidate 20-nucleotide single-guide RNA sequences targeting *SbMATL* (*Sobic.001G348600*), *SbDMP* (*Sobic.010G093200*), and *SbPLD3* (*Sobic.009G062600*) were designed using the CRISPR-P online tool (crispr.hzau.edu.cn). Two target sites were selected for *SbMATL* and one each for *SbDMP* and *SbPLD3* on the basis of two criteria: localization within exons and minimal predicted off-target effects (figs. S3, S7, and S10).

Each 20-nucleotide sequence was flanked by adapter sequences to generate forward primers (F) of the form 5′-AGATGATCCGTGGCA(N_20_)GTTTTAGCTATGC-3′, where “N_20_” represents the specific target sequence. Reverse primers (R) were complementary to their respective F primers (table S25). Primers were synthesized by Thermo Fisher Scientific and purified using polyacrylamide gel electrophoresis.

Forward and reverse primers were annealed in a 10-μl reaction containing 1 pmol of each primer. The mixture was denatured at 94°C for 10 min, cooled at 0.1°C/s to 15°C, and incubated for 10 min to complete annealing. Simultaneously, the PCas9-NPTII vector was linearized using *Aar I* restriction digestion. The linearized product was separated via agarose gel electrophoresis and purified using a Zymoclean Gel DNA Recovery Kit (Zymo Research).

Annealed oligonucleotides and linearized vectors were assembled via an In-Fusion HD Cloning Kit (Clontech) at a 1:3 molar ratio. The resulting *pCas9-SbMATL*, *pCas9-SbDMP*, and *pCas9-SbPLD3* constructs were transformed into *Escherichia coli* competent cells, and positive clones were selected on LB medium containing spectinomycin (50 mg/liter). Verified plasmids (confirmed by Sanger sequencing) were then transformed into the *Agrobacterium tumefaciens* strain EHA105 for plant transformation.

### Visual marker vector construction

To establish a visible marker for haploid identification, the RUBY expression cassette, conferring betalain pigmentation, was assembled into a binary vector containing a hygromycin resistance (*HPTII*) selectable marker. The resulting construct (pRUBY) was introduced into the *A. tumefaciens* strain EHA105 and subsequently used to transform embryogenic calli derived from the *Sbdmp-1 Sbmatl-2*;*P89GFP* (*NPTII*-free) inducer line.

Transformed T_0_ calli were selected on hygromycin-containing medium and screened for strong RUBY red pigmentation. Stable transformants exhibiting intense coloration were advanced to generate double-marker inducer lines designated *Sbdmp-1 Sbmatl-2*;*P89GFP-RUBY*.

### Production of transgenic plants

All binary vectors described above were introduced into the *A. tumefaciens* strain EHA105 by heat-shock transformation. Constructs containing *GFP* under the control of the 35*S* promoter and *RUBY* under the *Zea mays* ubiquitin promoter were included to generate sorghum plants expressing visible markers for haploid isolation. Single bacterial colonies carrying the verified constructs were sequence confirmed, cultured overnight in yeast extract beef medium, and stored as glycerol stocks at −80°C.

For plant transformation, immature embryos were used as explants. Developing seeds were harvested 11 to 13 days after flowering, carefully removed from the glumes, rinsed in 70% ethanol for 1 min, surface sterilized in 10% Clorox for 20 min, and rinsed four times with sterile distilled water. Embryos were dissected under a stereo microscope using fine forceps to minimize potential damage, centrifuged at 14,000 rpm for 1 min, and heat treated in a 42°C water bath for 3 min.

*Agrobacterium* cells were cultured overnight in yeast extract beef medium to an OD_600_ (optical density at 600 nm) of 1.0 to 1.2 and centrifuged at 8000 rpm for 3 min. The pellet was resuspended to an OD_600_ of 0.2 in liquid cocultivation medium [MS-CC; 1/10 MS salts, B5 vitamins, casamino acids (1 g/liter), proline (1 g/liter), asparagine (1 g/liter), maltose (30 g/liter), glucose (10 g/liter), 2,4-D (2 mg/liter), acetosyringone (200 μM); pH 5.2]. Embryos were incubated with the suspension for 10 min at room temperature, blotted dry on sterile Kimwipes, and transferred to solid MS-CC medium [solidified with agarose (7 g/liter)]. Cocultivation was conducted at 22°C for 3 days.

Embryos were then transferred to resting medium [MS-RE; MS salts, B5 vitamins, casamino acids (1 g/liter), asparagine (0.5 g/liter), sucrose (30 g/liter), sorbitol (10 g/liter), 2,4-D (2 mg/liter), 6-benzylaminopurine (0.2 mg/liter); pH 5.6] supplemented with timentin (200 mg/liter) and incubated at 28°C for 7 days. Subsequently, embryos were moved to selection medium (MS-SE) [MS-RE with timentin (200 mg/liter) and either paromomycin (50 mg/liter), hygromycin (5 mg/liter), or bialaphos (5 mg/liter), depending on the selectable marker]. Two rounds of selection (10 and 20 days each) were performed.

Callus surviving selection was transferred to shoot regeneration medium (MS-SR) [MS-SE supplemented with 6-benzylaminopurine (2 mg/liter) and naphthaleneacetic acid (0.1 mg/liter)]. After a 10-day subculture at 28°C under a 16-hour light/8-hour dark, calli were moved to selection-free MS-SR medium for 2 to 3 weeks. Regenerated shoots were transferred to rooting medium (MS-RT) [1/2 MS salts, B5 vitamins, sucrose (15 g/liter), and timentin (200 mg/liter); pH 5.6] containing the corresponding selective agent [paromomycin (50 mg/liter), hygromycin (5 mg/liter), or bialaphos (5 mg/liter)]. Rooted plantlets were transplanted to soil for genotyping. For the generation of double or triple knockout lines, transgene-free plants were obtained by segregation in progeny and retransformed with CRISPR-Cas9 vectors targeting additional genes of interest.

### Integration site and copy number determination of transgenes by genome resequencing

Genomic DNA was extracted from leaves of transgenic plants carrying the GFP marker using the cetyltrimethylammonium bromide method. DNA amplification and sequencing were performed according to standard protocols (*72*). Libraries were prepared for paired-end sequencing and run on an Illumina NovaSeq 6000 platform. Raw reads were filtered with fastp (version 0.12.4) to remove adaptors and low-quality sequences and aligned to the *S. bicolor* BTx623 reference genome (NCBI version 3.48) and T-DNA sequences using BWA-MEM (version 0.7.17) (*73*).

Insertion sites were identified from alignment data, and the copy number was determined from the number of insertion loci. Plants containing a single insertion were designated as single-copy events. Insertion sites were validated by polymerase chain reaction using primers flanking and within the T-DNA (table S25). Polymerase chain reactions contained Taq mix (Vazyme, P112-01), 20 ng of genomic DNA, and 0.2 μM of each primer. DNA from WT Tx430 or P898012 plants served as negative controls.

### Pollen viability and germination assays

To ensure the use of fresh pollens, we flicked off all anthers from flowering panicles in the evening just before the collection day. Pollens were flicked into a 1.5-ml Eppendorf tube at 8:00 to 9:00 a.m., with anthers removed with forceps, followed by immediate addition of either 0.5 ml of 1% I_2_/KI solution or 1% TTC (2,3,5-triphenyl tetrazolium chloride) solution. Pollens were collected from at least three panicles as replicates. Staining was performed for 5 min for starch staining and ~50 min for viability assay at room temperature. Stained pollens, together with solution, were pipetted into 60-mm petri dishes for microscopic observation. For quantification in the viability assay, we divided pollens into deeply, weakly, and not stained ones, representing high, medium, and low viability, respectively. Viability was calculated as the percentage of viable pollens over the total pollen observed.

For in vitro pollen germination assay, pollen collection was as described above, except that 50-ml tubes were used. The tubes were placed on ice immediately following pollen collection and then brought to the lab for a pollen germination ability test. After the removal of anthers and addition of 5 ml of liquid medium (50 μM KH_2_PO_4_, 10 mM CaCl_2_·2H_2_O, 0.01% H_3_BO_3_, 0.1% casamino acids, 13% sucrose, and 15% polyethylene glycol, molecular weight 4000), pollens were pipetted into 60-mm petri dishes and germinated at room temperature for 30 min. Germinating pollens were imaged using a Leica M305FCA fluorescence stereomicroscope with a DMC6200 camera and analyzed using ImageJ. Only pollens with clear tube growth were considered to have the germination ability. The germination ability was calculated as the percentage of germinating pollens over the total pollens observed.

### Pollen competition assay

Fresh pollens were collected as described above, except that paper bags were used instead of tubes. Pollens were measured with 1.5-ml Eppendorf tubes, mixed at a 1:1 ratio (0.2 ml of *Sbdmp Sbmatl inducer*:0.2 ml of WT), and then pollinated to the male-sterile line L407A. Pollination was repeated once the following day. Seeds were harvested 15 days after first pollination, grouped into normal and aborted classes according to their appearance, and separated again according to the presence or absence of the GFP signal. We assumed that all GFP-negative seeds were from fertilization of WT pollens and all GFP-positive seeds (either in embryos or in endosperms) were from fertilization of inducer pollens (possibly including heterofertilization as we cannot distinguish it in this experiment). Four panicles were used as replicates. The competition ability of the *Sbdmp Sbmatl* pollens was calculated as the percentage of GFP-positive seeds over total seeds sampled from a panicle.

### Identification of haploid embryos and plants

For isolation of immature haploid embryos (10 to 18 days postpollination), homozygous *GFP* inducer lines were used as male parents. Developing seeds were harvested, dehulled, and examined under a Leica M305FCA fluorescence stereomicroscope equipped with UV illumination. Embryos lacking GFP signals but surrounded by GFP-positive endosperms were classified as haploids, whereas embryos and endosperms both expressing GFP were identified as diploids. Aberrant embryos or endosperms, regardless of GFP expression, were also collected for analysis. When required, seeds were surface sterilized before dissection, and putative haploid embryos were germinated on half-strength MS medium containing sucrose (15 g/liter). Resulting seedlings were subjected to flow cytometry or whole-genome sequencing. For postharvest identification, mature dry seeds were germinated on wet filter paper and observed under a microscope. Seedlings lacking GFP fluorescence were designated haploids. Similarly, seeds from crosses with RUBY inducers were sown directly in soil, and nonpigmented seedlings were classified as haploids. Randomly selected haploid plants were verified by flow cytometry, genome sequencing, or morphological analysis.

### Ploidy determination by flow cytometry

Fresh leaf tissue (20 to 50 mg) was finely chopped with a razor blade in 1 ml of LB01 buffer [15 mM tris-HCl, 2 mM Na_2_EDTA, 0.5 mM spermine tetrahydrochloride, 80 mM KCl, 20 mM NaCl, 0.1% (v/v) Triton X-100, pH 7.5 adjusted with 1 M NaOH, and 15 mM β-mercaptoethanol]. The homogenate was gently pipetted, filtered through a 400-mesh nylon strainer, and supplemented with propidium iodide (100 μg/ml) and ribonuclease (100 μg/ml). Samples were incubated on ice for 10 min before analysis.

Nuclear fluorescence was quantified using a MoFlo XDP high-speed flow cytometer (Beckman Coulter Inc., US) equipped with a 70-μm ceramic nozzle and operated at 414-kPa sheath pressure. Propidium iodide fluorescence was excited using a 488-nm solid-state laser and collected with a 625 ± 26–nm high performance quality band-pass filter. The FL3-height/SSC (side scatter)-height gating method was used to exclude debris and nonnuclear material. Ploidy levels were determined on the basis of FL3-height/FL3-area ratios, where diploid nuclei corresponded to an FL3-area value of ~64 and haploid nuclei to ~32.

### Determination of genome origin in haploids by genome sequencing

Genomic DNA was extracted from leaves of the male-sterile line L407A, haploid inducer lines, and putative haploid embryos or leaves of putative haploid plants. To understand possible causes of malformed embryos or endosperms associated with HI at the genomic level, DNA was also extracted from those aberrant tissues. The embryo or endosperm of hybrid seeds produced using noninducer lines as the pollen donor was used as control. DNA amplification and sequencing were performed as described above. The samples were sequenced at ~5× depth, except that the female line and inducer lines were sequenced at a higher depth (30×) for better mining of single-nucleotide polymorphisms (SNPs) between parental lines. After filtration of the raw data with Fastp (version 0.12.4) to remove adaptors and low-quality reads, paired-end 150-bp reads of each sample were mapped to the BTx623 reference genome (NCBI version 3.48) using BWA with default parameters. The output files were sorted, with duplicates marked using sambamba (version 0.6.6), and then GATK (version 3.5.0) was employed for SNP calling. The low-quality SNPs (QUAL ≤20) were removed before downstream analysis. The genomic reference chromosomes were partitioned into consecutive nonoverlapping bins of 100,000 bp. For each bin in a given sample, the number of SNPs was recorded as either maternal or paternal origin by comparing the genotype of SNPs with parental lines. We use the genome origin index (GOI) to indicate the origin of a given bin, which is calculated as GOI = (number of SNPs of maternal origin − number of SNPs of paternal origin)/total SNPs in that bin. Using the embryo as an example, if GOI is one or close to one, it suggests a haploid genome of maternal origin; if GOI is around zero, it suggests a diploid genome of biparental origin. If there are less than five SNPs in a bin, it is ignored, and GOI is set as “NA” and not plotted.

### Induction of temporal male sterility by TFMSA

To enable cross-pollination with haploid inducer lines, temporal male sterility was induced using TFMSA, following a modified version of a previously reported protocol (*51*). TFMSA was dissolved in distilled water at 30 mg/ml, with optional additives—glycerol (5%), Tween 20 (0.25%), or both. Instead of foliar application, the TFMSA solution (10 to 15 mg per plant) was pipetted directly into the whorl of young leaves at 15 to 80 days after planting.

Treatment effectiveness was monitored by examining anther size and pollen sterility under a dissection microscope. Plants were then pollinated with GFP-expressing lines to determine the proportion of hybrid seeds, confirming successful emasculation. The optimized TFMSA treatment protocol was subsequently applied to produce female parents for large-scale crosses with haploid inducer lines.

### Evaluation of spontaneous chromosome doubling and field management

To evaluate the spontaneous chromosome doubling capacity, haploid plants were grown under field conditions in Beijing, with panicles bagged before anthesis to prevent unintended pollination. The seed set was measured at physiological maturity, and plants were categorized into four classes on the basis of seeds per panicle: no doubling (0 seeds), low (0.01 to 5%), medium (5 to 10%), and high (>10%). Female fertility was assessed via controlled pollination of haploid plants with WT pollen donors. For genotypes exhibiting low spontaneous doubling efficiency, we increased tillers by replanting plants at low density and with more intense field management (fertilization, disease and pest control, and weed control). Furthermore, haploid seedlings initially grown in Beijing were transplanted to Hainan (Yazhou, 109°17′E, 18°36′N) during winter to take advantage of favorable environmental conditions. Environmental stability was assessed by comparing doubling efficiency between Beijing and Hainan. Statistical analyses were conducted in R (version 4.1.2) using generalized linear models to evaluate the effects of genotype, environment, and their interaction on doubling efficiency.

### Chromosome doubling by colchicine treatment

The L407A male-sterile plants or TFMSA-emasculated F_1_ crosses were pollinated with inducer pollens. We used 116 haploid embryos isolated 15 days after pollination, of which 40 embryos were treated with 0.005% colchicine and 40 embryos with 0.01% colchicine, with 36 untreated ones as a control. The treatment was conducted on the hormone-free half-strength MS medium (1.5% sucrose) for 10 hours. The embryos were then germinated on the same medium without the addition of colchicine for 17 days. In parallel, diploid embryos were also germinated as another control. The third fully expanded leaf was cut off from individual plants for flow cytometry analysis. One diploid plant, 5 from untreated haploid embryos, 11 from 0.005% colchicine-treated haploid embryos, and 11 from 0.01% colchicine-treated haploid embryos were sampled. Flow cytometry analysis was performed essentially the same as described above.
