# Supplementary material for: Stepwise construction of the path to doubled haploid breeding in sorghum
Source: Sci Adv. 2026 May 22;12(21):eaed5464. doi: 10.1126/sciadv.aed5464 (PMC13196752; doi:10.1126/sciadv.aed5464)
Supplement: Supplementary file 1 — Figs. S1 to S30 Legends for tables S1 to S25 [file sciadv.aed5464_sm.pdf]

Science Advances
AAAS

Supplementary Materials for

Stepwise construction of the path to doubled haploid breeding in sorghum

Yi Sui *et al.*

Corresponding author: Yi Sui, suiyi@caas.cn; Sanyuan Tang, sytang@genetics.ac.cn;
Chuanyin Wu, wuchuanyin@caas.cn

Sci. Adv. **12**, eaed5464 (2026)
DOI: 10.1126/sciadv.aed5464

The PDF file includes:

Figs. S1 to S30
Legends for tables S1 to S25

Other Supplementary Material for this manuscript includes the following:

Tables S1 to S25

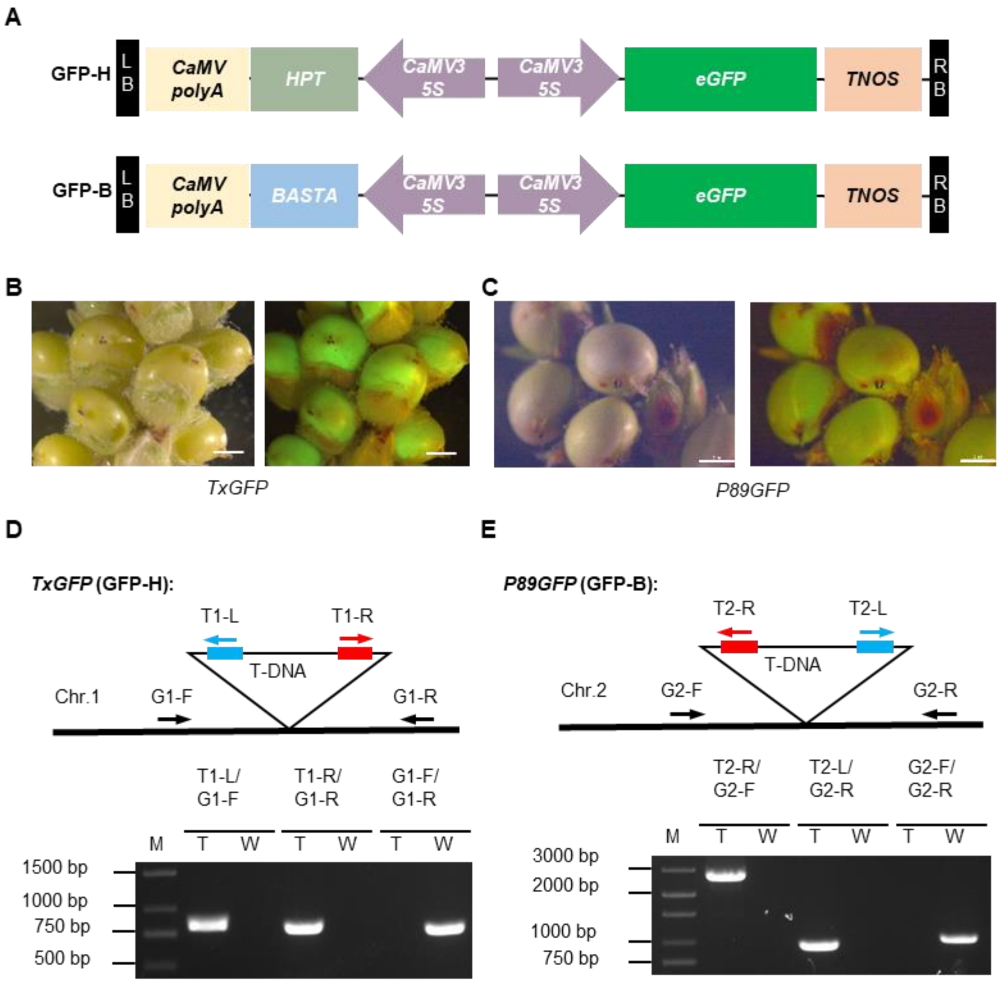

Fig. S1. **Development of efficient single-copy fluorescent marker lines in sorghum (Tx430 and P898012 backgrounds).**

(**A**) Schematic diagram of the GFP-H and GFP-B binary vectors used for *Agrobacterium-mediated* transformation. (**B** and **C**) Stable GFP fluorescence in T_2 seeds of the *TxGFP* (B) and *P89GFP* (C) lines under white light (left) and UV light (right). (**D** and **E**) Validation of single-copy T-DNA insertion sites. *TxGFP* (D) carried GFP-H inserted into Chromosome 1 (Chr1: 4,617,987), while *P89GFP* (E) carried GFP-B inserted on Chromosome 2 (Chr2: 75,331,531). Black arrows indicate genome-flanking primers, and red (T-R) and blue (T-L) arrows indicate T-DNA border primers. Electrophoresis confirmed the correct insertion sites. W, wild-type; T, *TxGFP* or *P89GFP* lines; M, molecular marker ladder; red rectangle, the right border of T-DNA; blue rectangle, the left border. Scale bars: 2 mm (B and C).

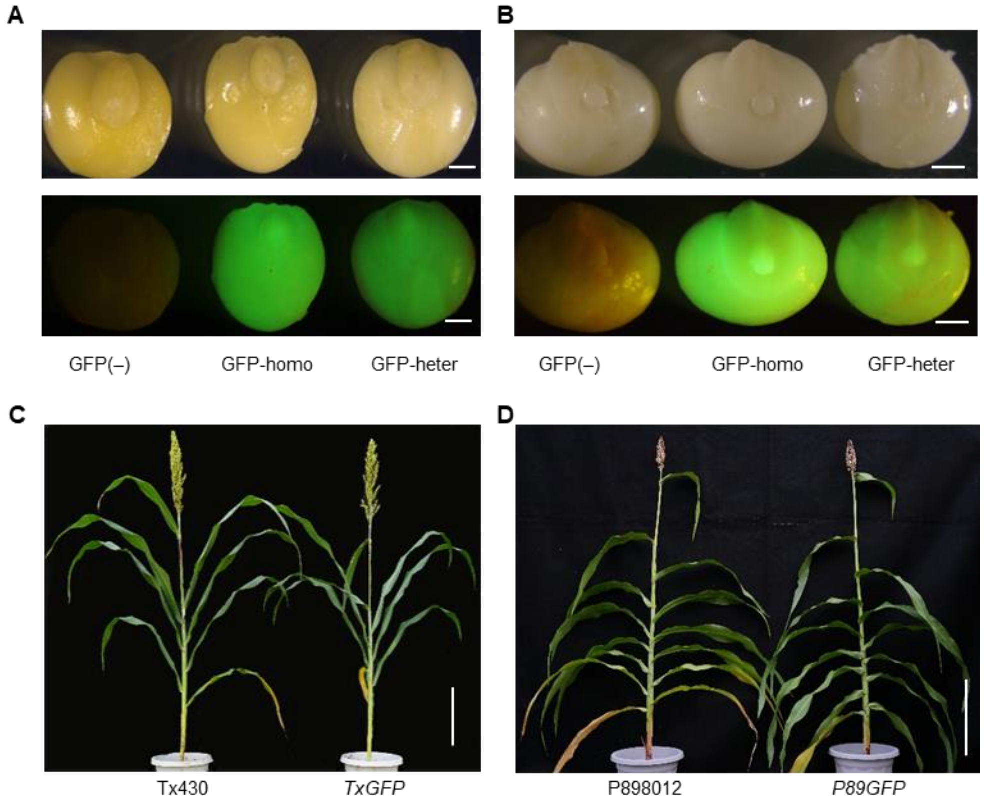

Fig. S2. **Validation of GFP marker lines for distinguishing haploid and diploid embryos.** (**A** and **B**) Comparison of fluorescent signal strength among wild-type (GFP–), homozygous GFP (GFP-homo), and heterozygous GFP (GFP-heter) seeds derived from crosses of the male-sterile line L407A × *TxGFP* (A) or L407A × *P89GFP* (B). Seed coats were removed for visualization. (**C** and **D**) Phenotypic comparison of *TxGFP* (C) and *P89GFP* (D) lines relative to their wild-type backgrounds. Scale bars: 0.5 mm (A), 1 mm (B), 2 mm (C and D).

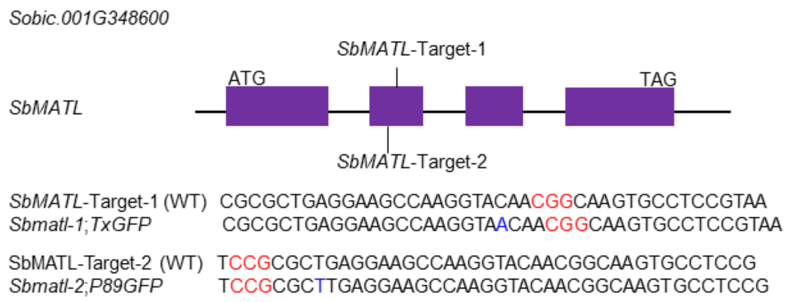

Fig. S3. CRISPR/Cas9-induced mutations in the *SbMATL* gene.

Schematic of the *SbMATL* gene structure showing target sites and corresponding loss-of-function mutations in two genetic backgrounds. PAM sequences are highlighted in red, guide RNA sequences are emphasized, and insertion mutations are indicated in blue.

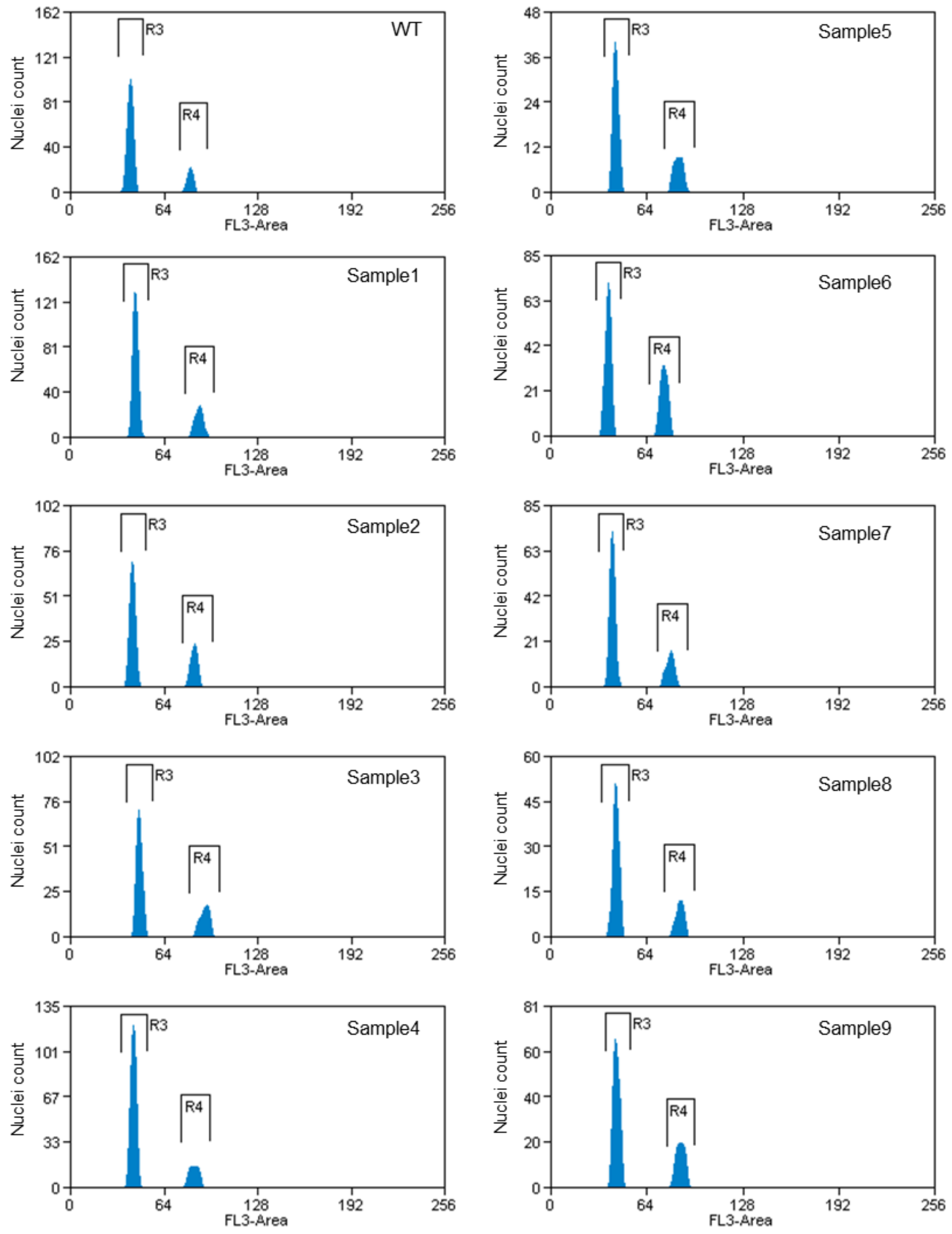

Fig. S4. **Flow cytometry verification of diploid wild-type plants.**

Representative histograms of nine wild-type (WT) samples showing consistent diploid peaks. The x-axis indicates nuclear fluorescence intensity; the y-axis represents the number of nuclei analyzed.

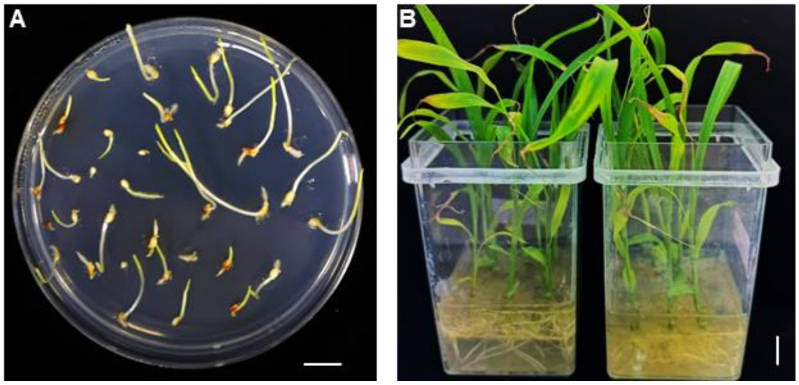

Fig. S5. **Germination and early development of haploid embryos.**

(**A**) Putative haploid embryos germinating on hormone-free MS medium. (**B**) Plants germinated from embryos at the early shoot stage. Scale bars: 10 mm (A), 2 cm (B).

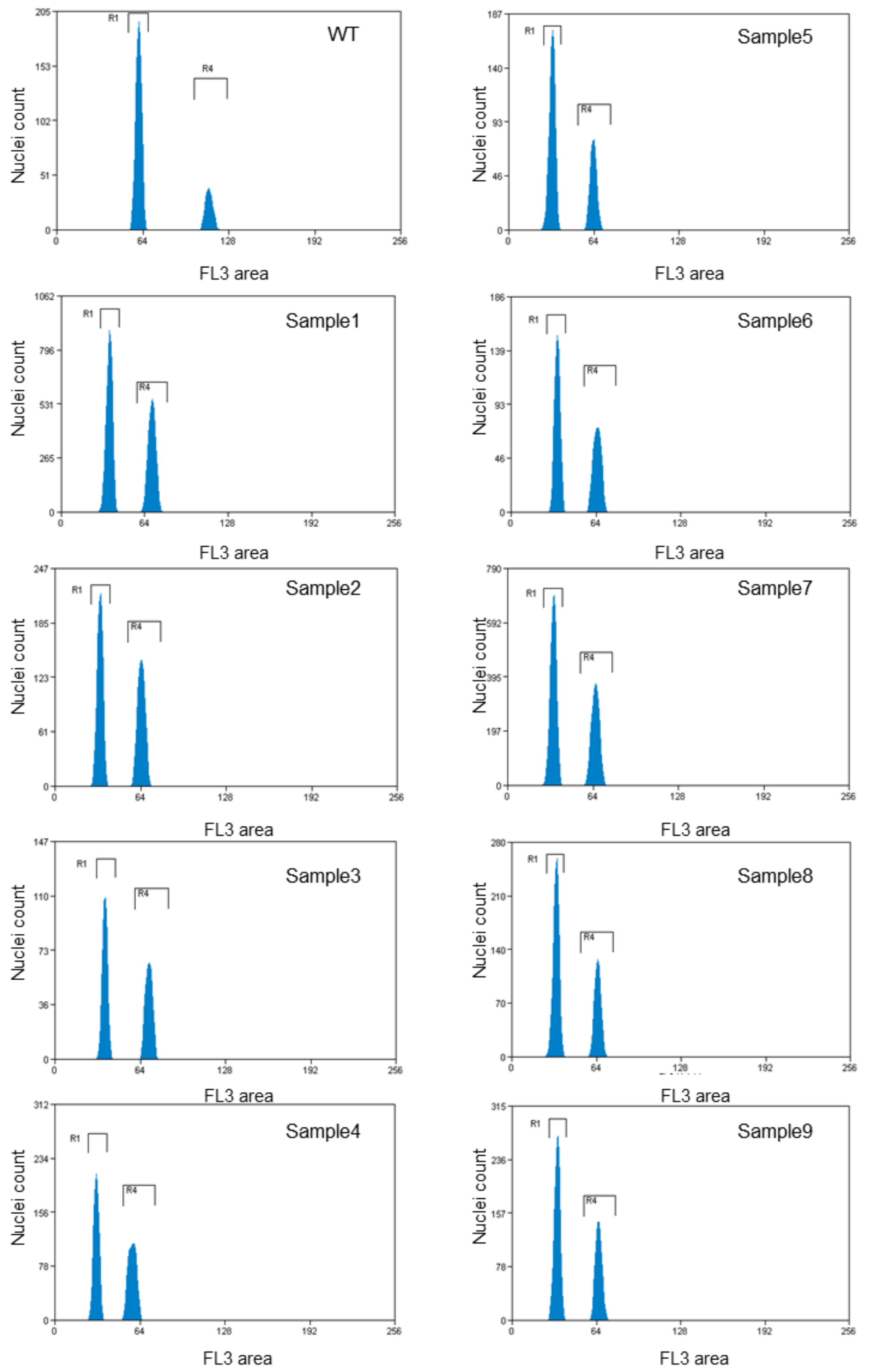

Fig. S6. **Flow cytometry confirmation of haploids from *Sbmatl* inducers.**

Representative analysis of nine putative haploids compared with wild-type (WT) L407A plants. The x-axis indicates nuclear signal intensity, and the y-axis represents the number of nuclei.

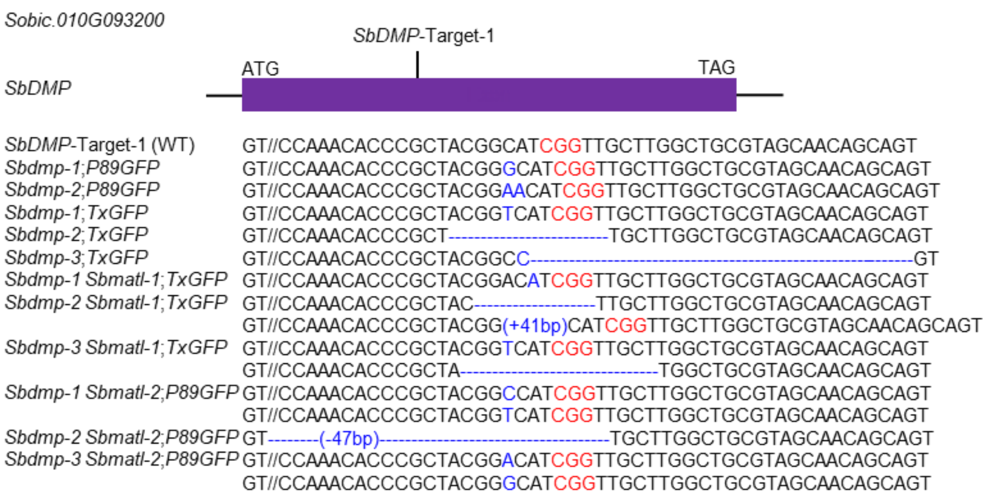

Fig. S7. **CRISPR/Cas9-induced mutations in the *SbDMP* gene.**

Schematic showing *SbDMP* gene structure, CRISPR target sites, and mutation types in two genetic backgrounds. PAM sequences are in red; guide RNA sequences are emphasized. Insertions appear in blue, deletions as blue dashes.

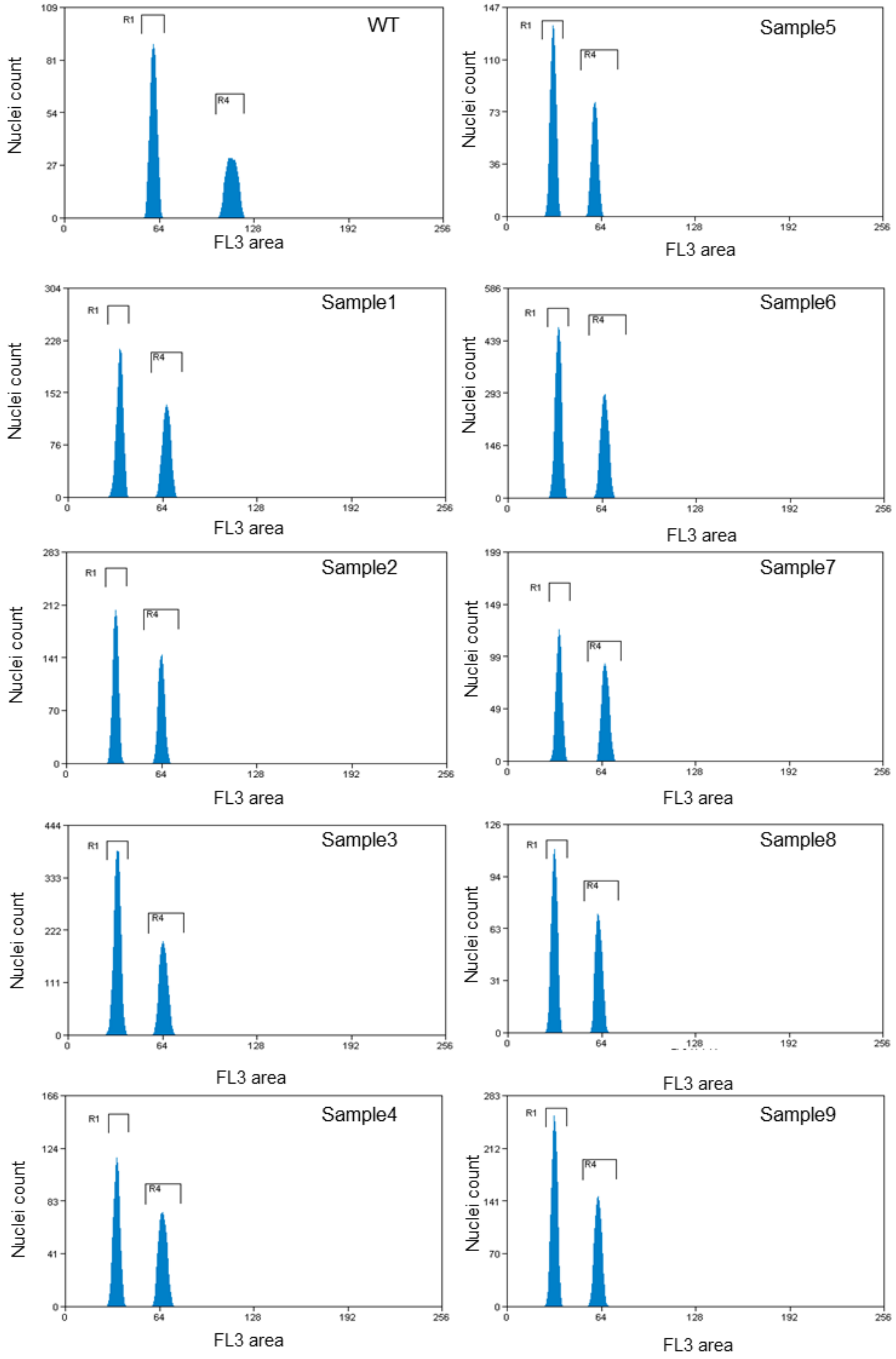

Fig. S8. **Flow cytometry confirmation of haploids induced from *Sbmatl Sbdmp* double mutants.**

Representative analysis of nine haploids compared with wild-type (WT) L407A controls. The x-axis indicates the nuclear signal peak; the y-axis indicates the number of nuclei.

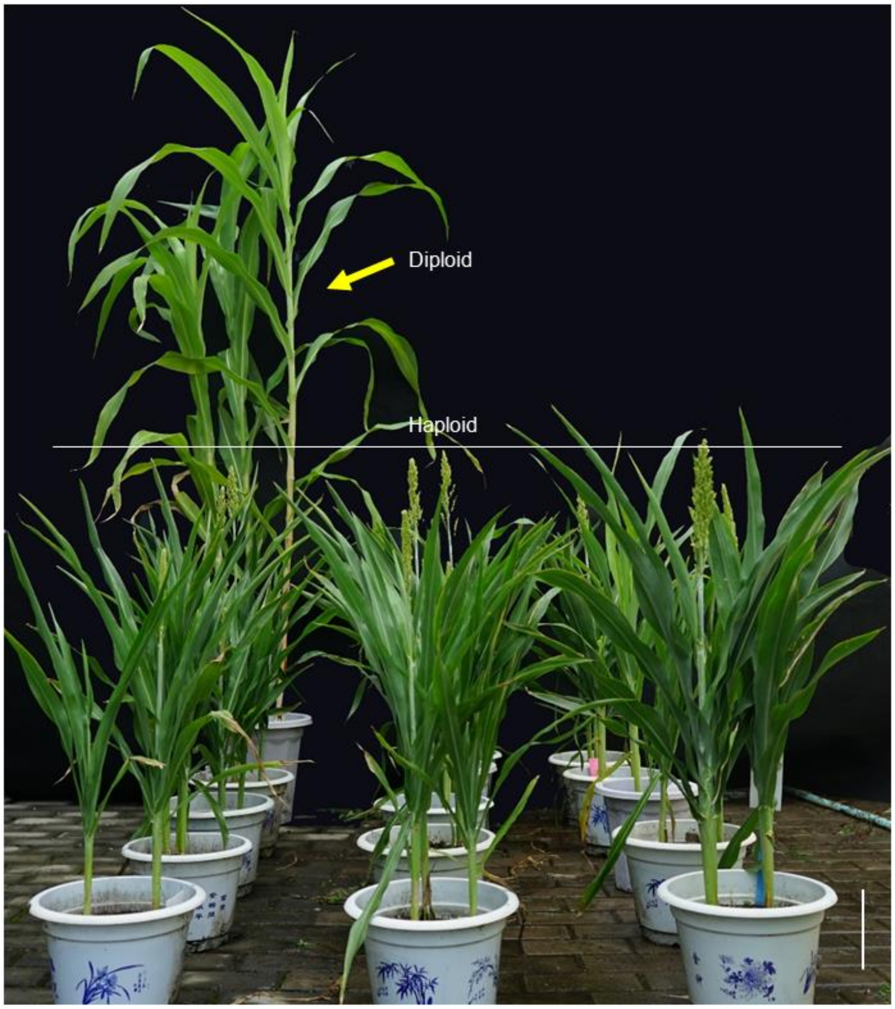

Fig. S9. **Morphological comparison of maternal haploid and hybrid diploid (F_1) plants.** Haploids (white line) exhibit smaller vegetative and reproductive organs than their diploid F_1 counterparts (yellow arrow). Scale bar, 20 cm.

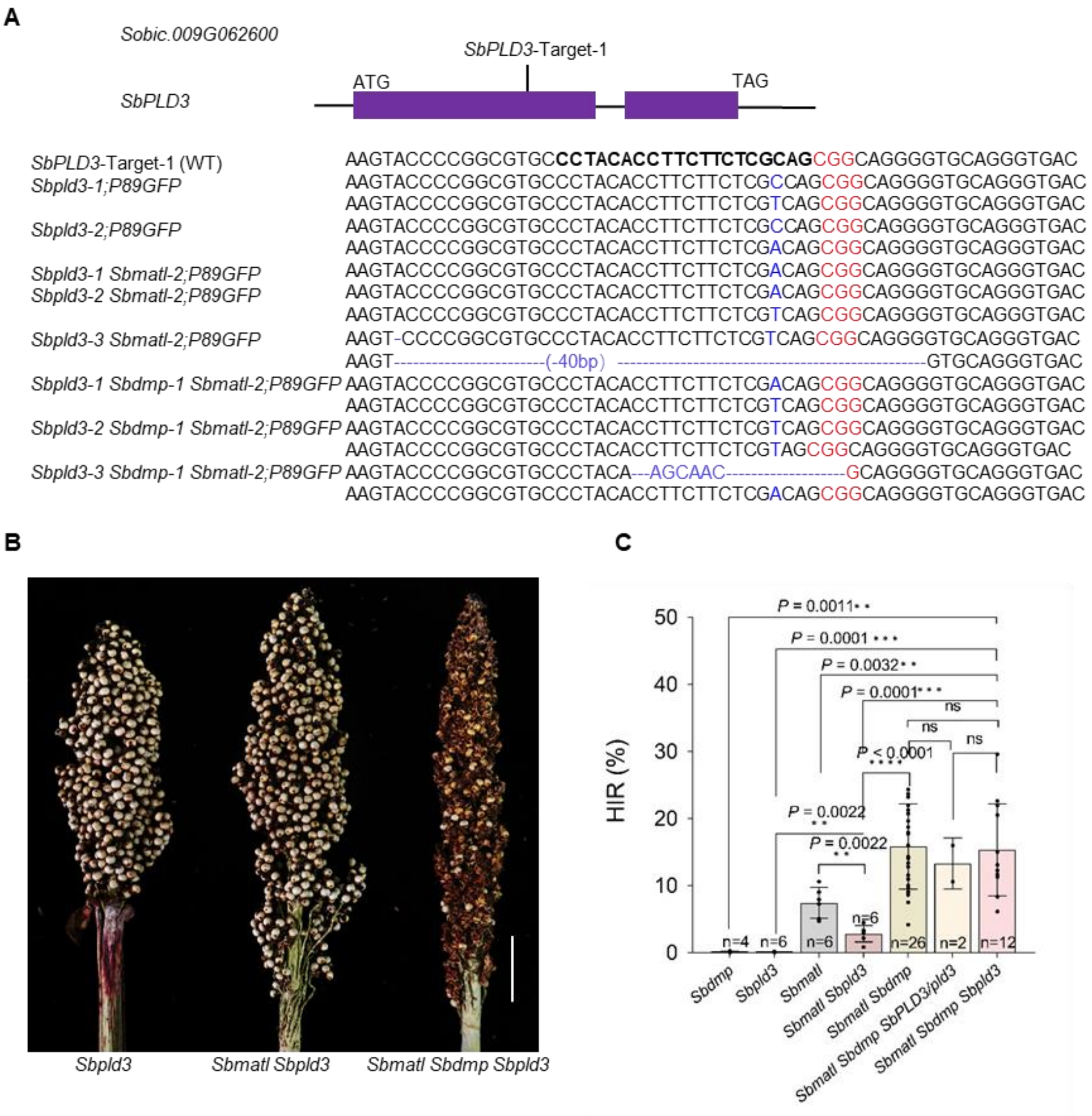

Fig. S10. ***SbPLD3* mutation does not enhance haploid induction in sorghum.**

(**A**) *SbPLD3* gene structures showing CRISPR/Cas9 target sites and mutation types in the *P89GFP* background. PAM sites are in red; guide RNAs are emphasized; insertions are in blue; deletions are shown as blue dashes. (**B**) Panicle phenotypes of *Sbpld3*, *Sbmatl Sbpld3*, and *Sbmatl Sbdmp Sbpld3* mutants. Scale bar, 2 cm. (**C**) HIR of L407A pollinated by *Sbdmp*, *Sbpld3*, *Sbmatl*, *Sbmatl Sbpld3*, *Sbmatl Sbdmp*, *Sbmatl Sbdmp SbPLD3*/*pld3*, and *Sbmatl Sbdmp Sbpld3*/*pld3* mutants under Beijing pot conditions. Error bars represent mean ± s.d.; significance determined by two-sided Mann-Whitney test (**$p < 0.01$, ***$p < 0.001$, ****$p < 0.0001$).

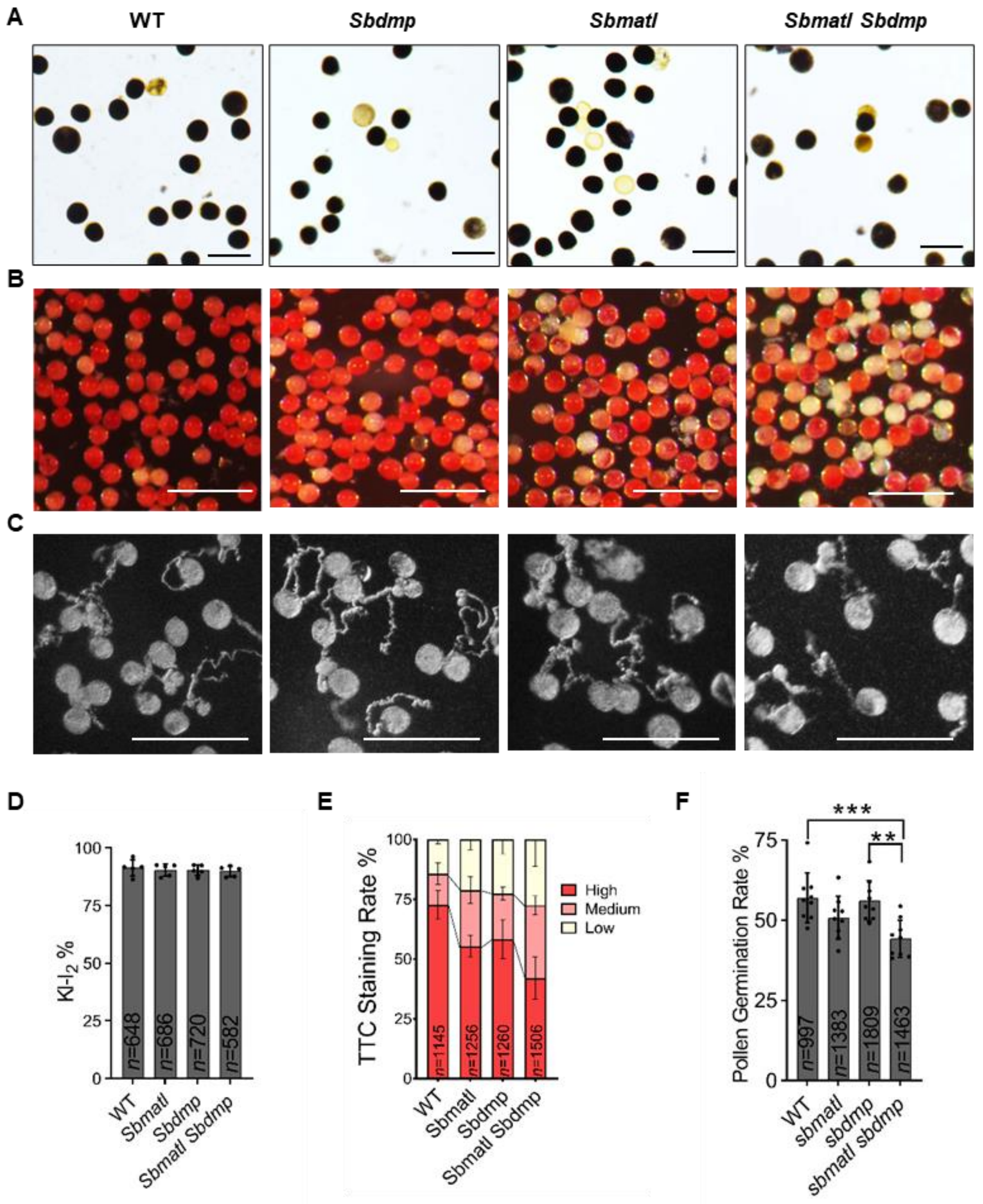

Fig. S11. Comparative evaluation of pollen fertility, viability, and germination in wild-type, *Sbmtl*, *Sbdmp*, and *Sbmtl Sbdmp* double mutants.

(**A**) Assessment of pollen fertility using KI/I_2 staining. (**B**) Measurement of pollen viability via TTC staining. (**C**) Evaluation of pollen germination on germination medium. (**D** to **F**) Statistical analysis of pollen fertility (D), viability (E), and germination (F) percentages from (A) to (C), respectively (n = 5 fields of view for D and E, n = 9 for F). Error bars represent mean ± s.d.; significance determined by two-sided Mann-Whitney test (***p* < 0.01, ****p* < 0.001). Scale bars: 100 μm (A), 200 μm (B and C).

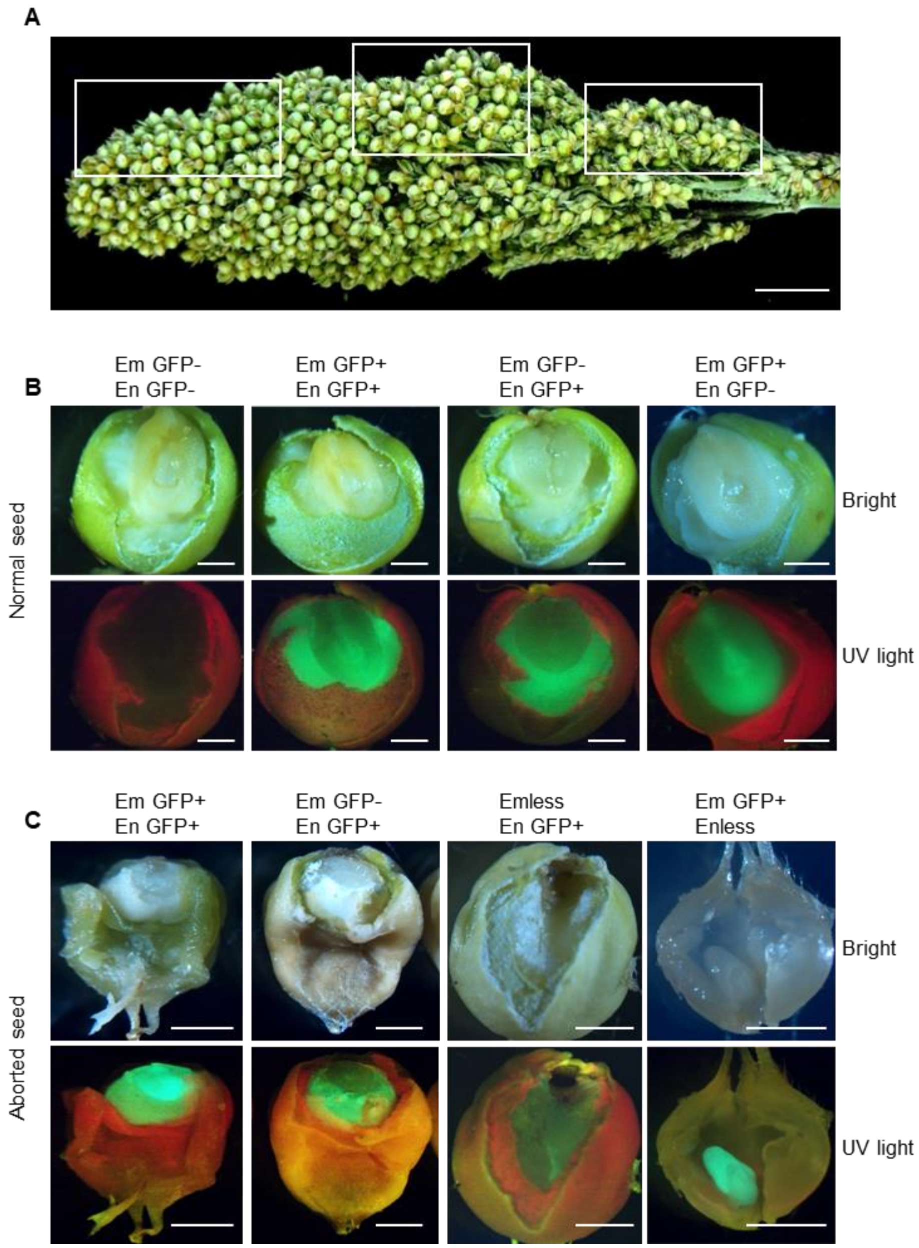

Fig. S12. Pollen competition assay between inducer and WT plants.

(**A**) A representative panicle harvested 15 days after pollination with pollens mixed at a 1:1 ratio between inducer and WT plants. Seeds were sampled from top, middle, and low portions (indicated with rectangles) for pollen competition assessment. (**B**) Seeds of normal appearance were grouped into 4 classes based on presence or absence of GFP signals in embryo or endosperm. (**C**) Aborted seeds were also grouped into 4 classes. Em, embryo; En, endosperm; Emless, lack of embryo; Enless, lack of endosperm. Scale bars: 2 cm (A), 1 mm (B and C).

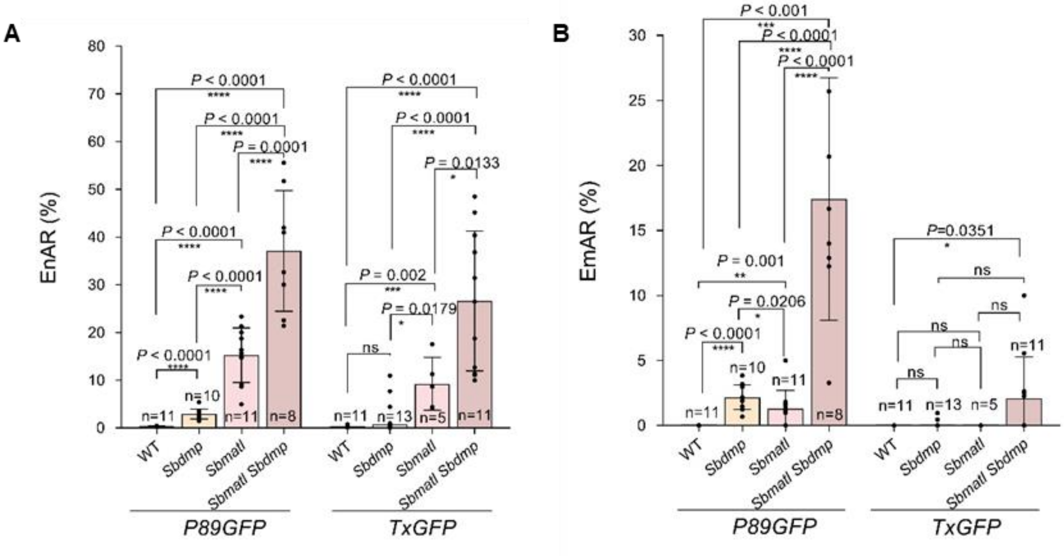

Fig. S13. **Comparison of endosperm abortion rate (EnAR) and embryo abortion rate (EmAR) among three mutants in two sorghum backgrounds.**

(**A** and **B**) Bar plots showing EnAR (A) and EmAR (B) of S*bmatl*, *Sbdmp*, and *Sbmatl Sbdmp* mutants along with wild-type (*TxGFP* and *P89GFP*). Error bars denote means ± s.d.; ns: not significant, *$p < 0.05$, **$p < 0.01$, ***$p < 0.001$, ****$p < 0.0001$ (two-sided Mann–Whitney test). n = number of panicles analyzed.

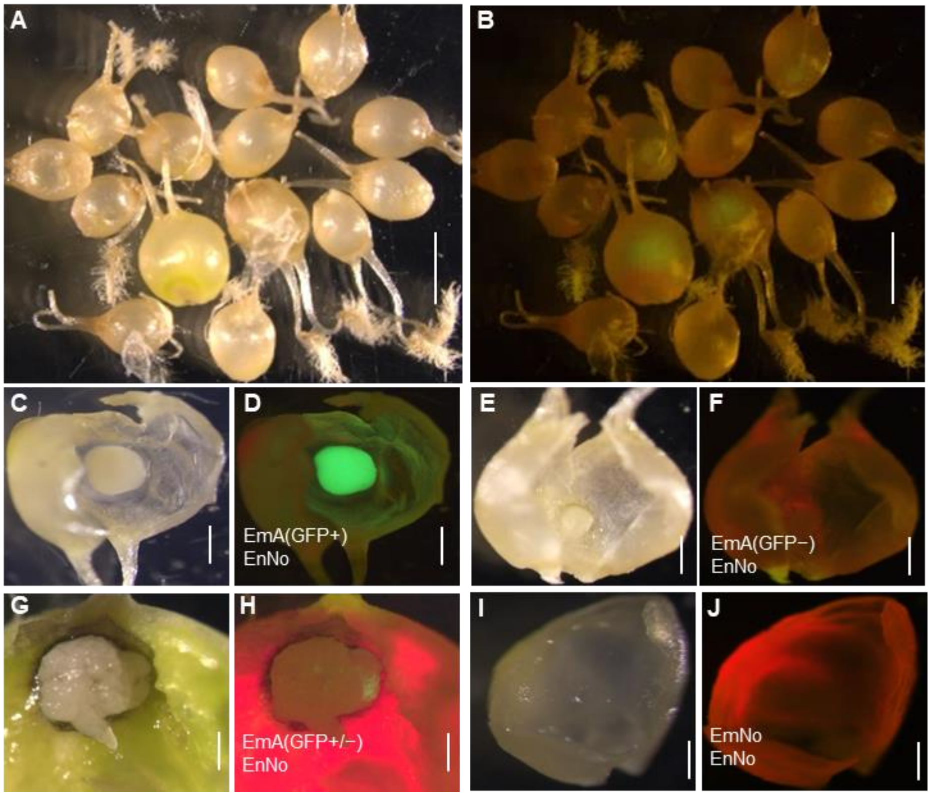

Fig. S14. **Representative embryos arrested in cross pollinated florets.**

(**A** and **B**) Fertilized or unfertilized embryo sacs 14 days after pollination under white (A) and UV (B) light. EmA, abnormal embryo, EnNo, lack of endosperm. (**C** to **H)** Embryo sacs showing developmental arrest, with (D, H) or without (F) GFP expression. (**I** and **J**) Vesicular structures of empty embryo sacs. Scale bars: 2 mm (A and B), 0.5 mm (C to F), 0.3 mm (G and H), 250 µm (I and J).

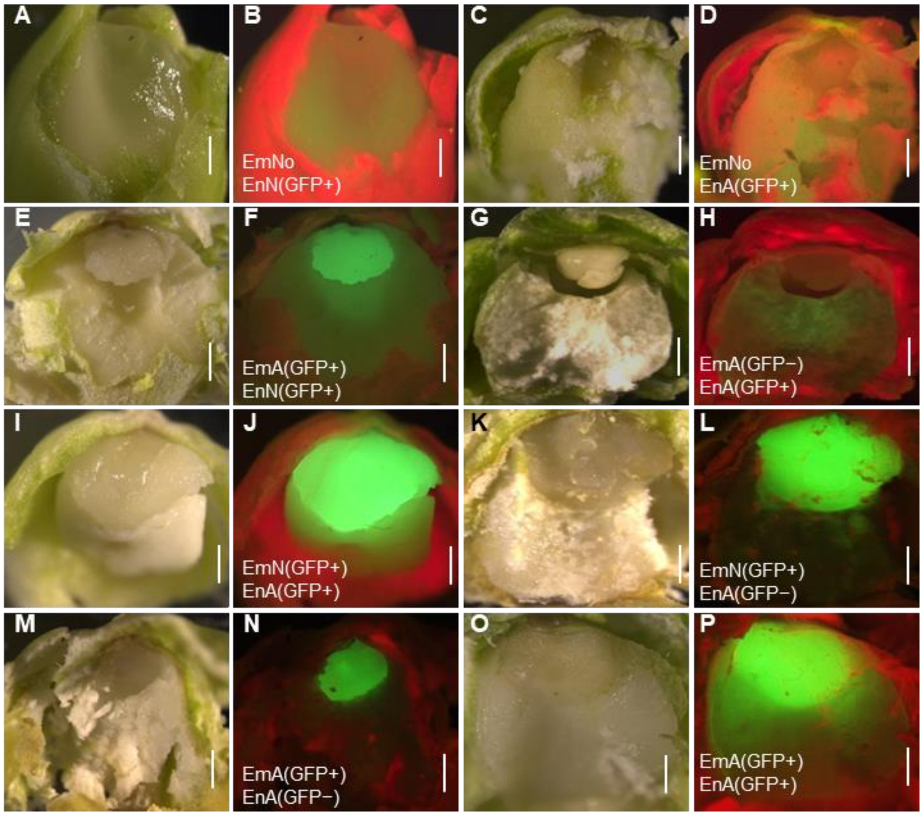

Fig. S15. **Morphological types of aborted seeds after cross-pollination. with inducer lines.** (**A** to **D**) Embryo-less seeds (EmNo) with normal (A and B) or aborted (C and D) endosperm. (**E** to **H**) Malformed embryos (EmA) with normal endosperm (EnN) or malformed endosperm (EnA) under white and UV light. (**I** to **L**) Seeds showing malformed endosperm (EnA) but normal embryos (EmN). (**M** to **P**) Seeds with both a malformed embryo and endosperm. Scale bar, 0.5 mm.

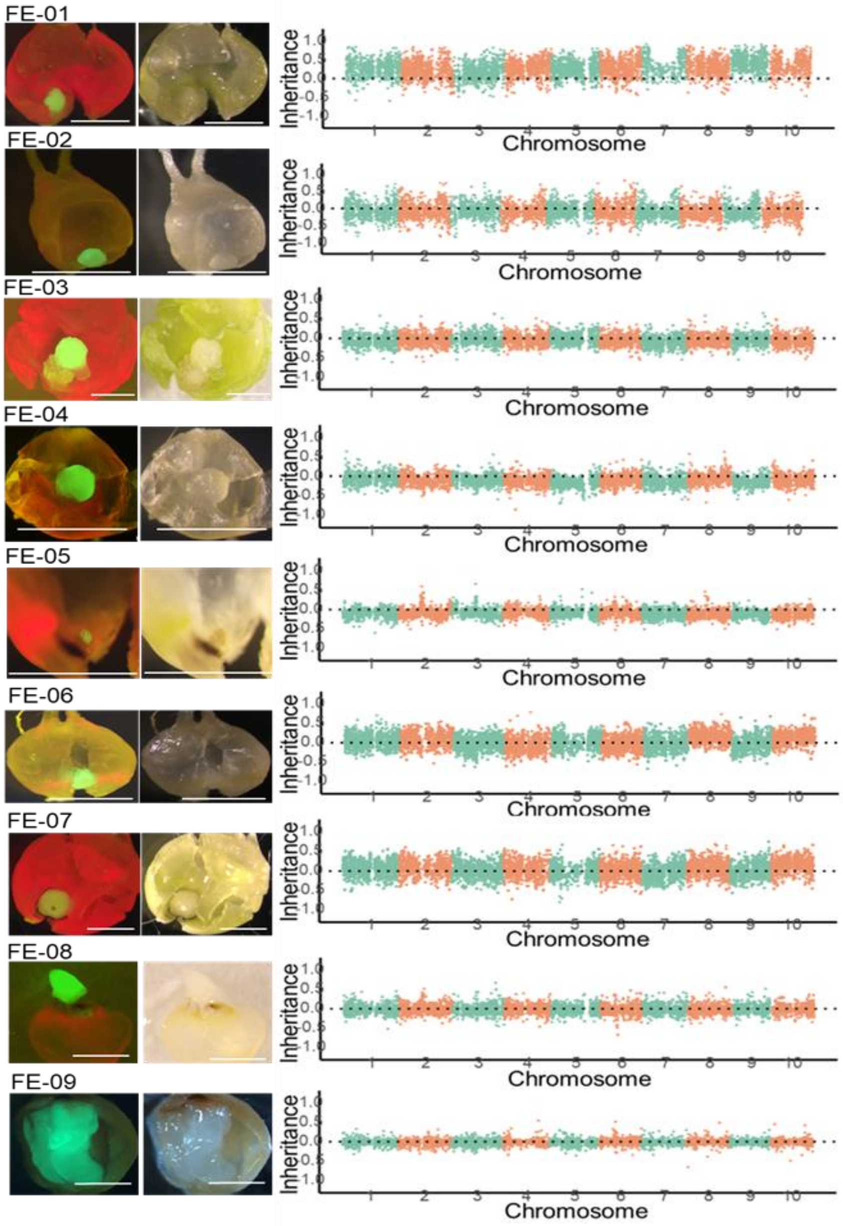

Fig. S16. Single fertilization of the egg cell results in defective seed development.
Pearl-like miniature embryos with GFP signals following pollination by inducer lines (left panel), suggesting single fertilization of the egg cell. Whole-genome sequencing confirmed the single fertilization of the egg cell by a sperm cell (bi-parental genome origin), accompanied possibly by failed fertilization of the central cell, resulting in the absence of endosperm. Subsequent embryonic arrest occurred due to a lack of endosperm support. FE, fertilized embryo. Scale bar, 1 mm.

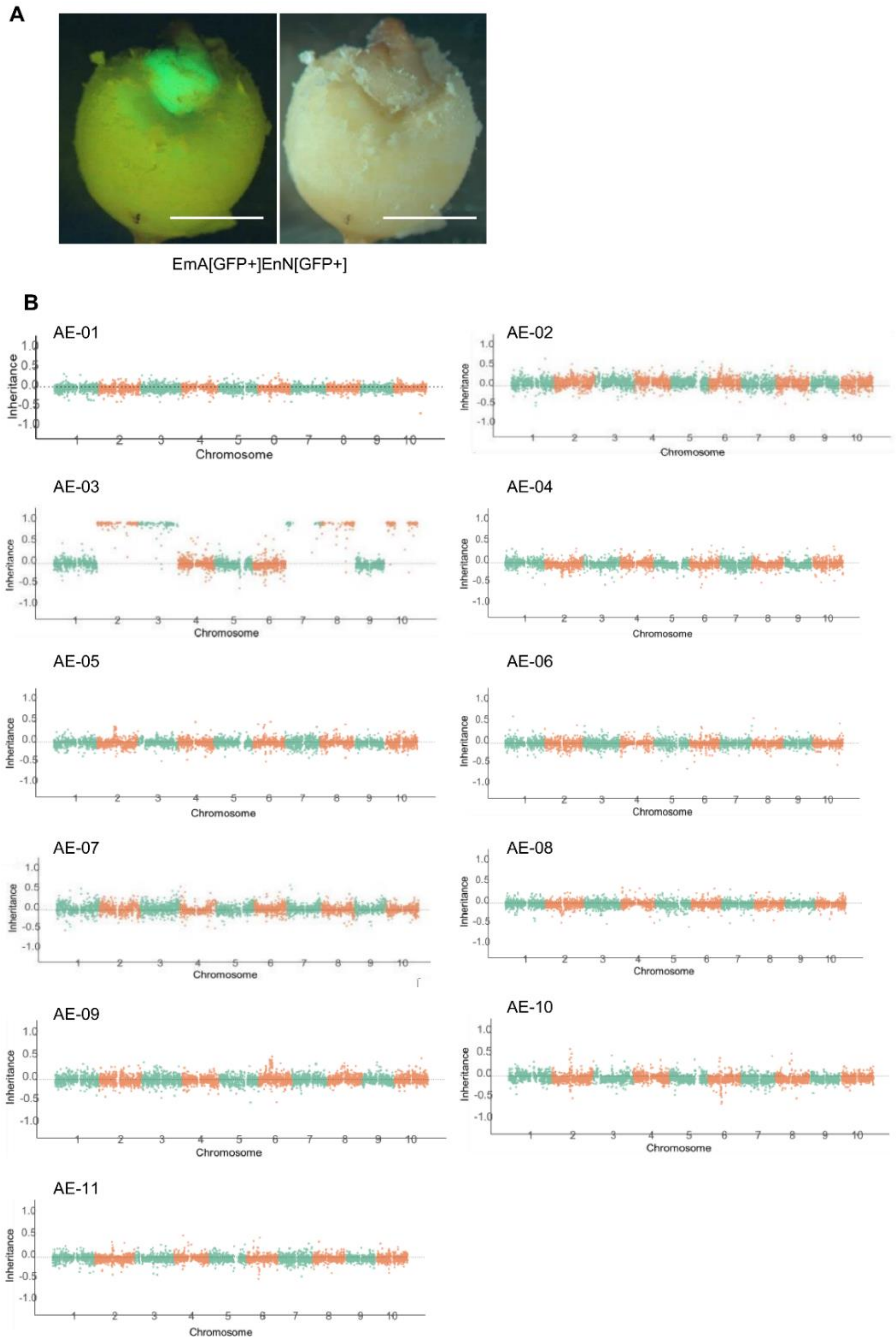

Fig. S17. Genome characterization of aborted embryos.

(**A**) A representative aborted embryo exhibiting GFP signal in the normal endosperm after pollination by inducer lines. (**B**) Whole-genome sequencing confirms biparental origin in most samples, with one (AE-03) showing loss of specific paternal chromosomal segments (Chr 2, 3, 7, 8, 10). AE, aborted embryo. Scale bar, 1 mm.

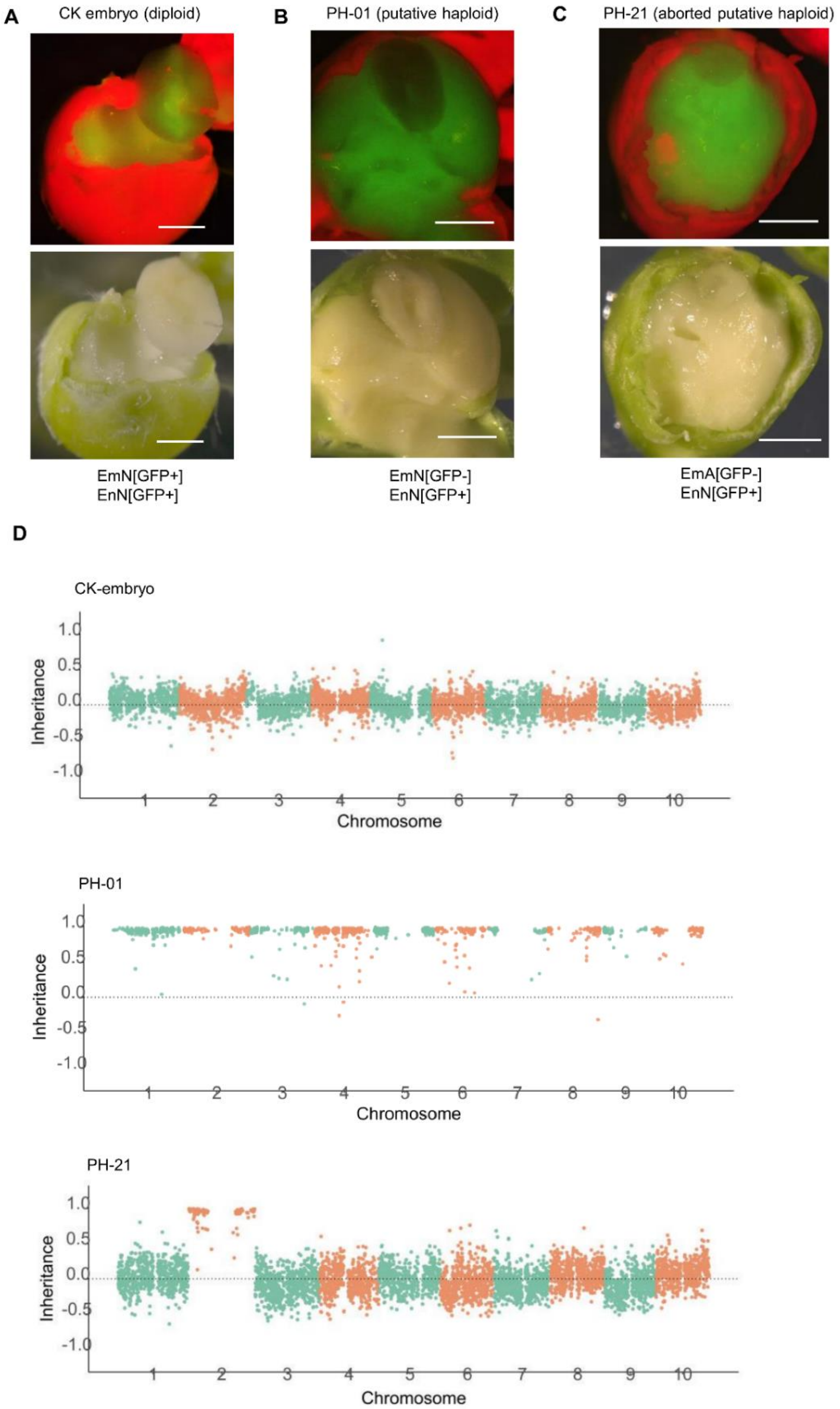
A
CK embryo (diploid)
EmN[GFP+]
EnN[GFP+]
B
PH-01 (putative haploid)
EmN[GFP-]
EnN[GFP+]
C
PH-21 (aborted putative haploid)
EmA[GFP-]
EnN[GFP+]
D
CK-embryo
PH-01
PH-21
Inheritance
1.0
0.5
0.0
-0.5
-1.0
Chromosome
1
2
3
4
5
6
7
8
9
10

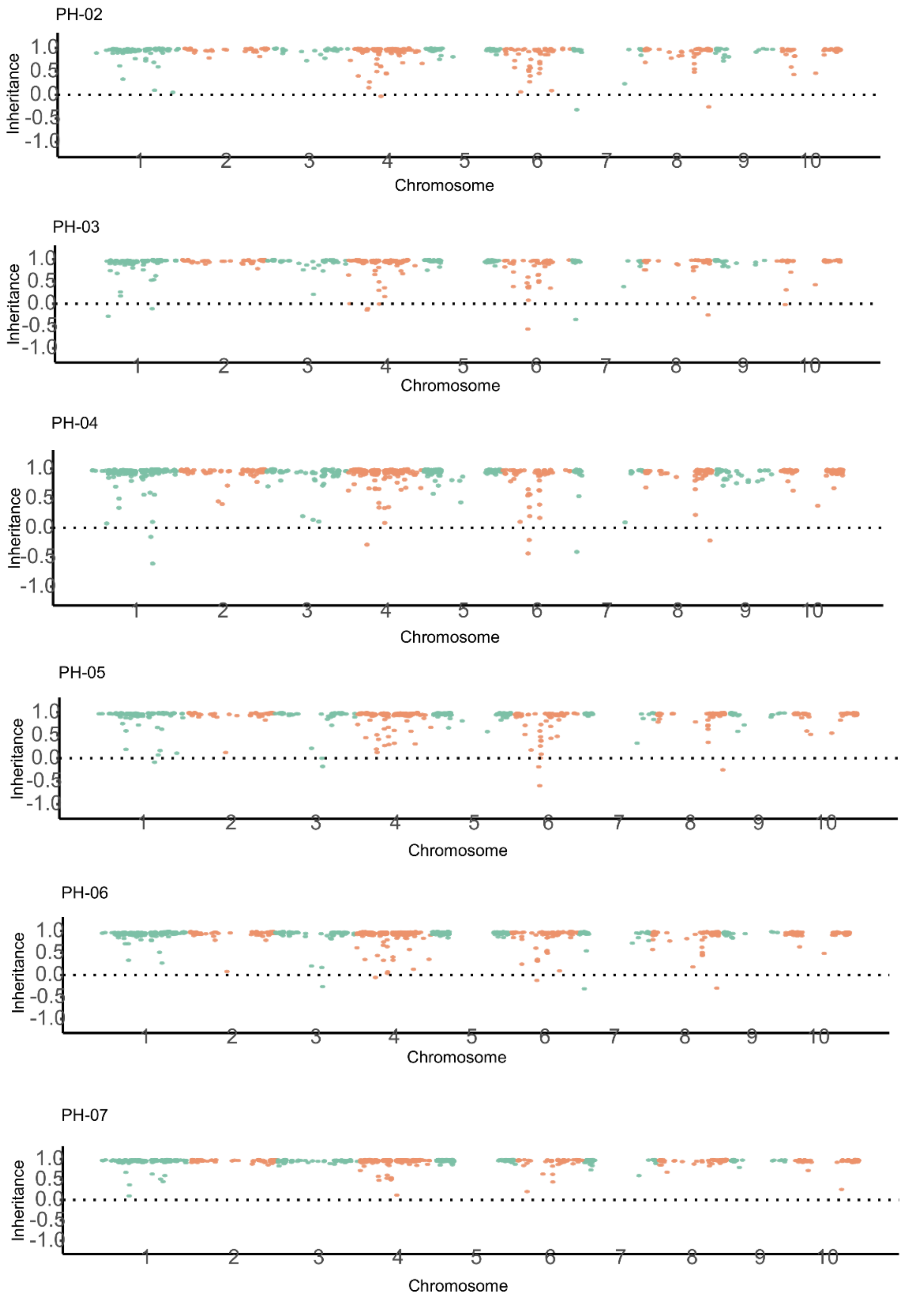
PH-02
Inheritance
1.0
0.5
0.0
-0.5
-1.0
1
2
3
4
5
6
7
8
9
10
Chromosome
PH-03
Inheritance
Chromosome
PH-04
Inheritance
Chromosome
PH-05
Inheritance
Chromosome
PH-06
Inheritance
Chromosome
PH-07
Inheritance
Chromosome

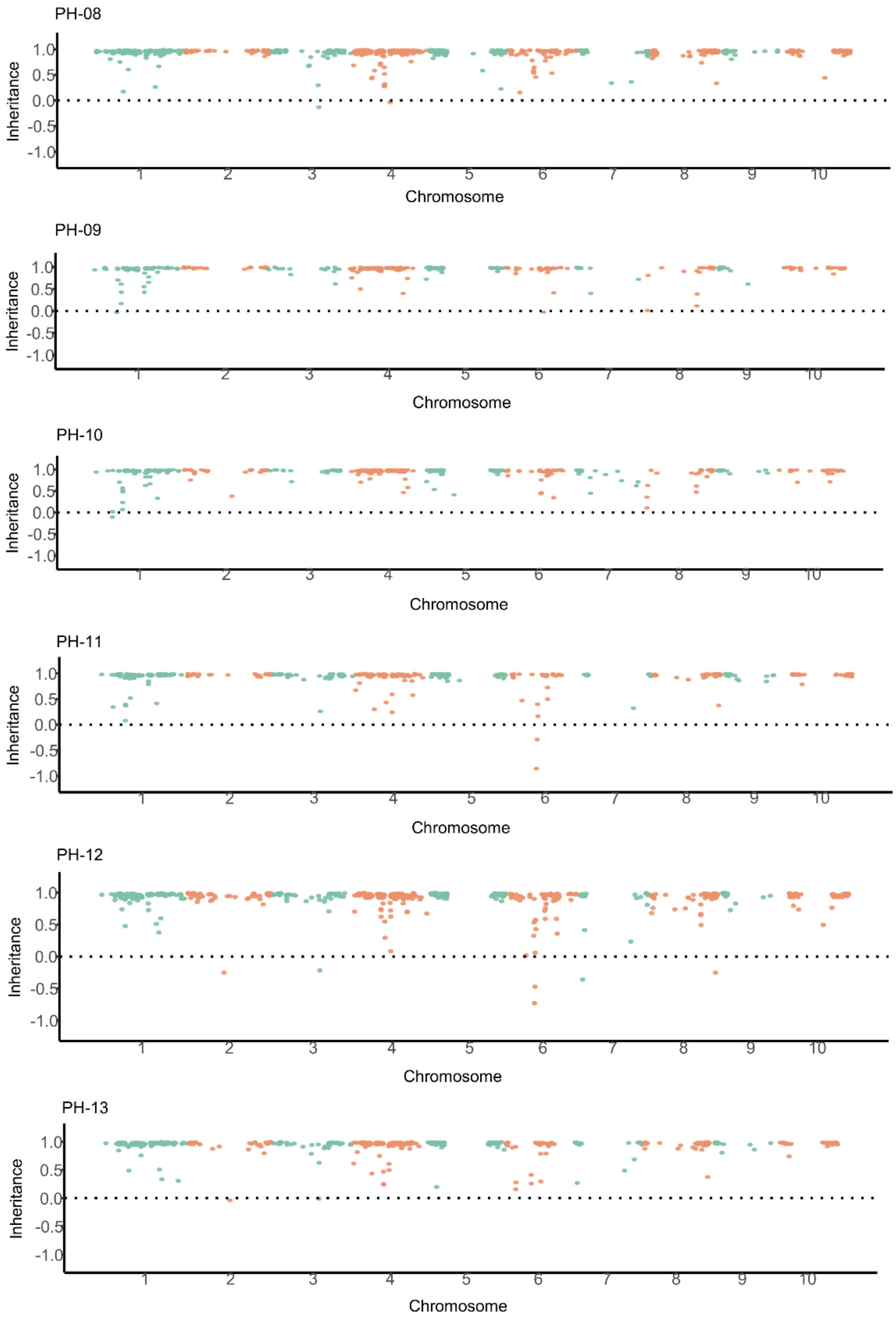
PH-08
PH-09
PH-10
PH-11
PH-12
PH-13
Inheritance
1.0
0.5
0.0
-0.5
-1.0
1 2 3 4 5 6 7 8 9 10
Chromosome

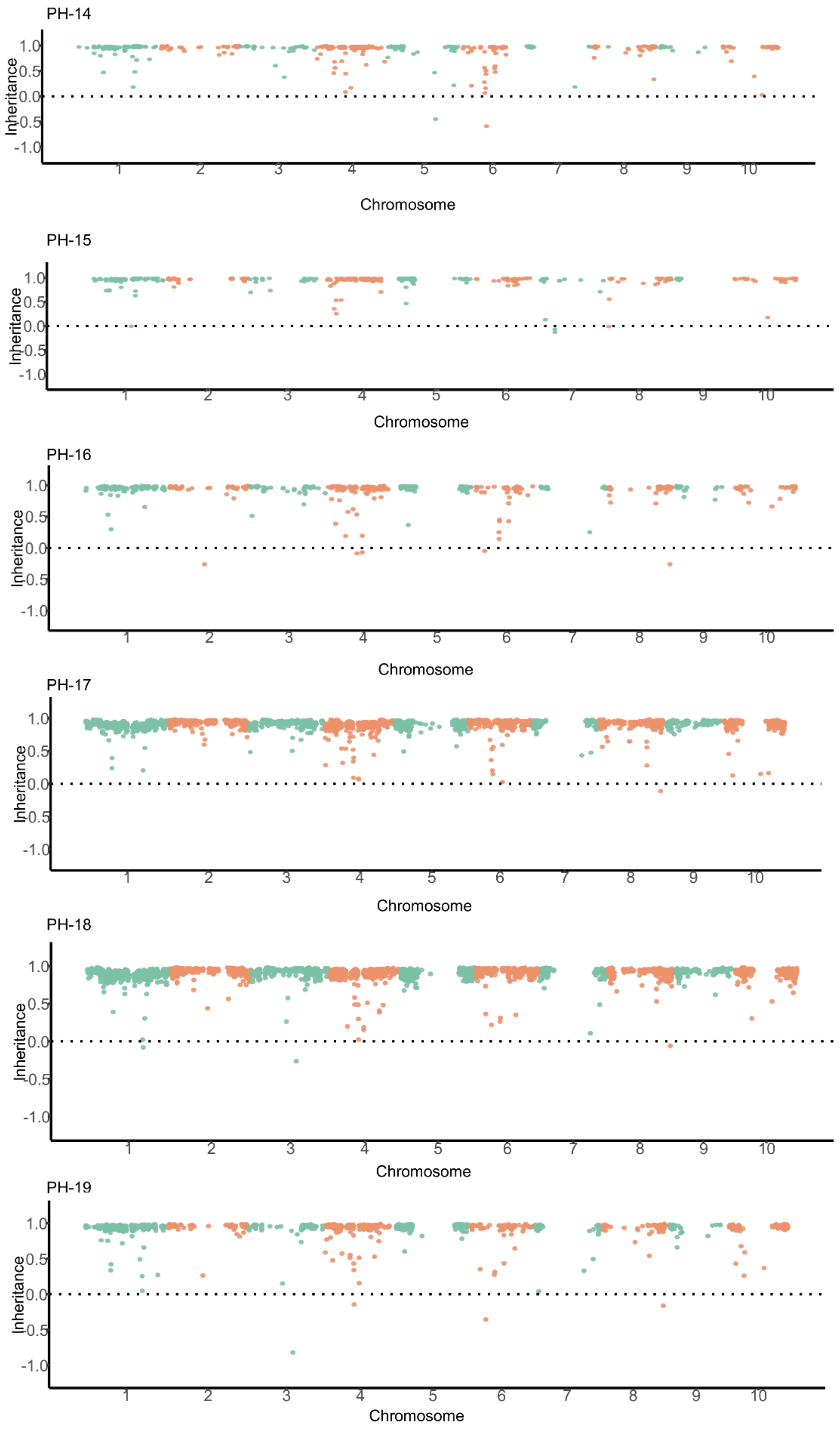
PH-14
Inheritance
1.0
0.5
0.0
-0.5
-1.0
1 2 3 4 5 6 7 8 9 10
Chromosome
PH-15
Inheritance
1.0
0.5
0.0
-0.5
-1.0
1 2 3 4 5 6 7 8 9 10
Chromosome
PH-16
Inheritance
1.0
0.5
0.0
-0.5
-1.0
1 2 3 4 5 6 7 8 9 10
Chromosome
PH-17
Inheritance
1.0
0.5
0.0
-0.5
-1.0
1 2 3 4 5 6 7 8 9 10
Chromosome
PH-18
Inheritance
1.0
0.5
0.0
-0.5
-1.0
1 2 3 4 5 6 7 8 9 10
Chromosome
PH-19
Inheritance
1.0
0.5
0.0
-0.5
-1.0
1 2 3 4 5 6 7 8 9 10
Chromosome

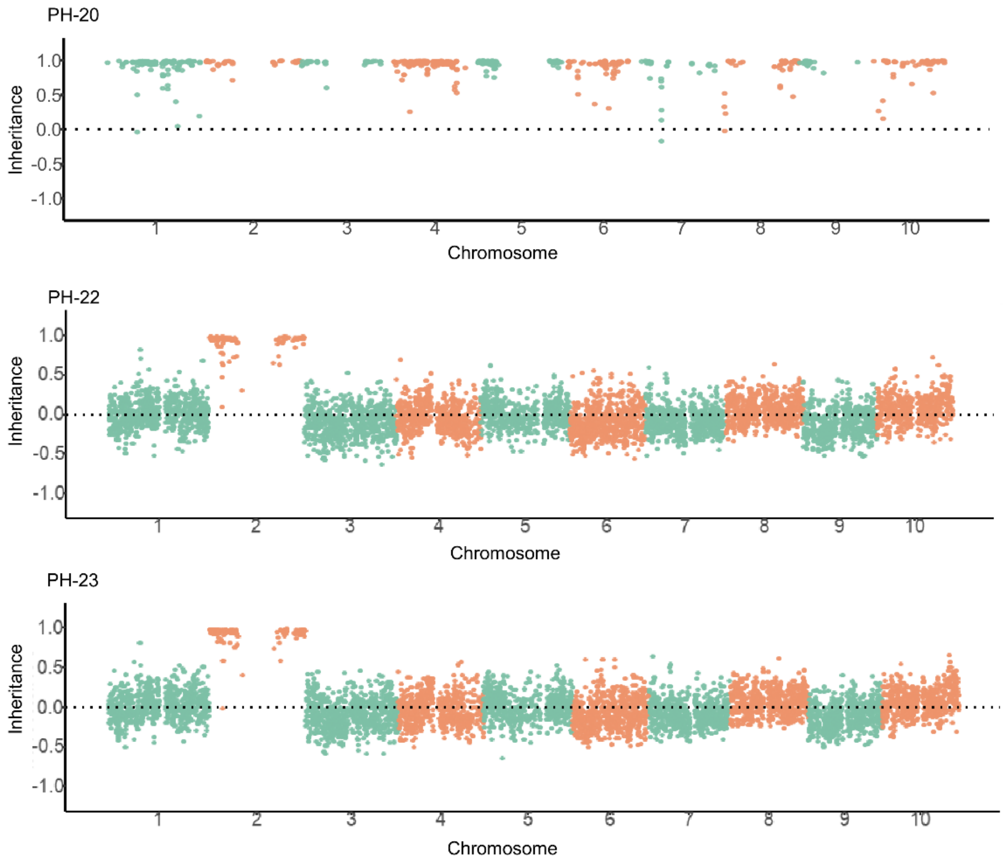

Fig. S18. Genomic identification of putative haploid (PH) embryos.

(**A**) Diploid hybrid seed expressing GFP in both embryo and endosperm (EmN[GFP^+]EnN[GFP^+]). (**B**) Typical putative haploid seed showing GFP^- embryo with GFP^+ endosperm (EmN[GFP^-]EnN[GFP^+]). (**C**) Aborted putative haploid embryo lacking GFP, paired with GFP^+ endosperm (EmA[GFP^-]EnN[GFP^+]). (**D**) Whole genome sequencing validation of putative haploids. CK-embryo: diploid control (as in A); PH-1 to 20: haploid embryo lacking paternal chromosomes (as in B); PH-21 to 23: embryos lacking paternal chromosome 2, which harbors the GFP T-DNA insertion (as in C). Scale bars: 1 mm (A to C).

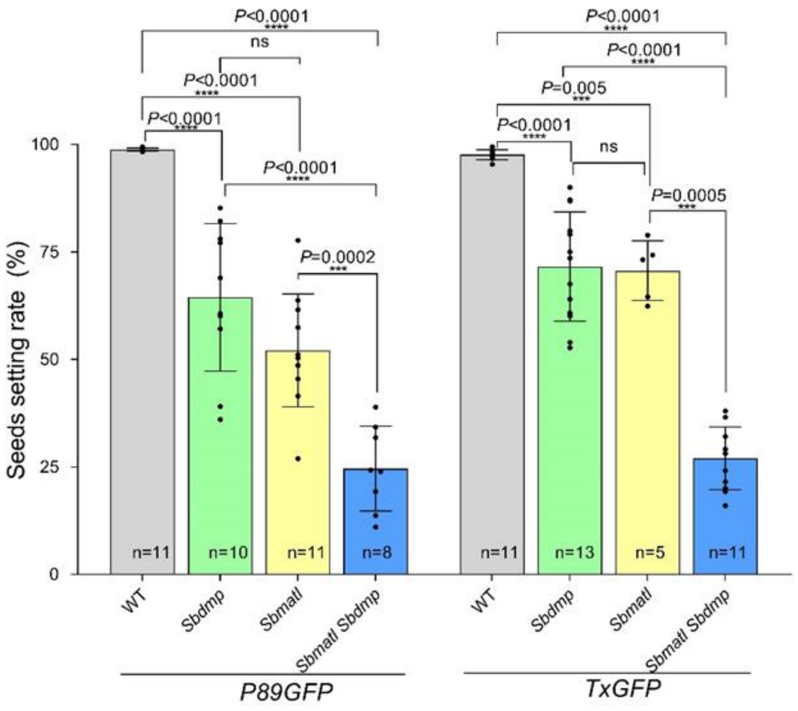

Fig. S19. Reduced seed-set rate in *Sbmatl*, *Sbdmp,* and *Sbmatl Sbdmp* mutants. Quantitative comparison of seed-set percentages relative to wild-type in *P89GFP* and *TxGFP* backgrounds. Error bars denote means ± s.d.; ns: not significant, ***p < 0.001, ****p < 0.0001 (two-sided Mann–Whitney test). n = number of panicles analyzed.

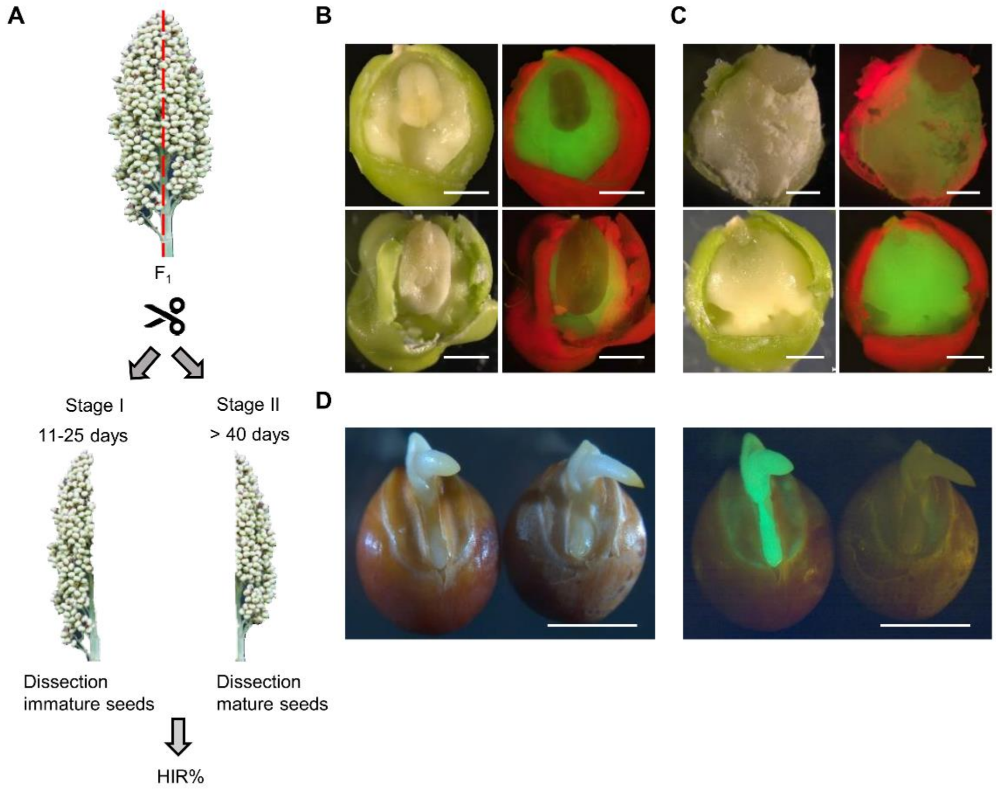

Fig. S20. **Timeline of haploid identification and developmental following cross-pollination.** (**A**) Schematic of sampling stages: immature seeds (11–25 days after pollination, stage I) and mature seeds (>40 days, stage II). (**B** and **C**) Haploid embryos screening under white and UV illumination, showing normal haploids lacking GFP in the embryo (B) and abnormal haploids with defective embryo and endosperm (C). (**D**) Germination assays distinguishing diploid hybrids (GFP^+ shoots) from haploids (GFP^- shoots with GFP^+ endosperm) under white (left) and UV (right) illumination. Scale bars: 1 mm (B and D), 0.5 mm (C).

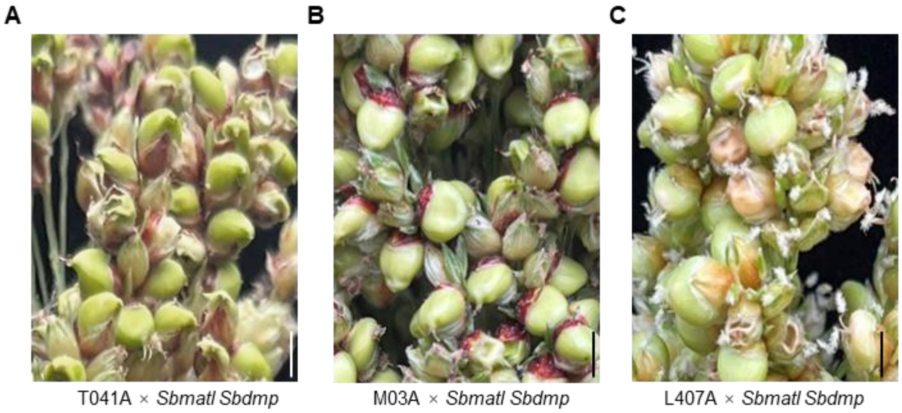

Fig. S21. **Seed sets in crosses of three male-sterile lines with inducer line.**

(**A** to **C**) Panicle phenotypes of male-sterile lines T041A (A), M03A (B), and L407A (C) pollinated by *Sbmatl Sbdmp* inducer. Scale bar: 5 cm.

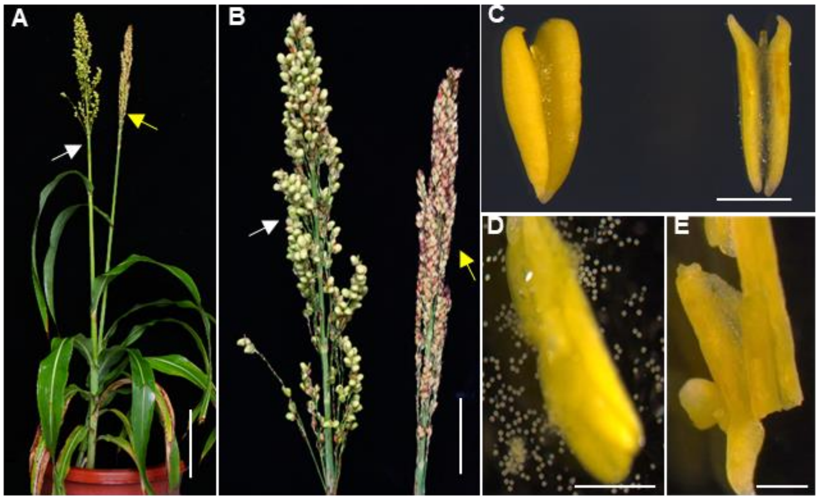

F

Maternal parent	Paternal parent	Total hybrid seeds	Total Germinated Haploid seeds	HIR (%)
F_1 (ZH0639/ZH0612）	*Sbmatl Sbdmp;P89GFP*	401	42	10.47
F_1 (D070/D055)	*Sbmatl Sbdmp;P89GFP*	466	34	7.30
F_1 (ZH0617/D095)	*Sbmatl Sbdmp;P89GFP*	278	18	6.47
F_1 (ZH0617/D075)	*Sbmatl Sbdmp;P89GFP*	482	29	6.02
F_1 (ZH0617/D070)	*Sbmatl Sbdmp;P89GFP*	589	51	8.66
F_1 (D092/ZH0612)	*Sbmatl Sbdmp;P89GFP*	763	102	13.37

Fig. S22. **Trifluoromethanesulfonamide (TFMSA)-induced male sterility and its application in haploid induction.**

(**A**) TFMSA treatment produced male sterility (yellow arrows) compared to untreated fertile panicles (white arrows). (**B**) A close-up view of panicles in A. (**C**) Fertile anther (left) versus sterile anther after TFMSA treatment (right). (**D** and **E**) Pollen release from untreated (D) and sterile (E) anthers. (**F**) Six elite lines treated with TFMSA produced haploids when crossed with *Sbmatl Sbdmp; P89GFP* or *Sbmatl Sbdmp; TxGFP* inducers. Scale bars: 10 cm (A), 5 cm (B), 1 mm (C), 500 µm (D and E).

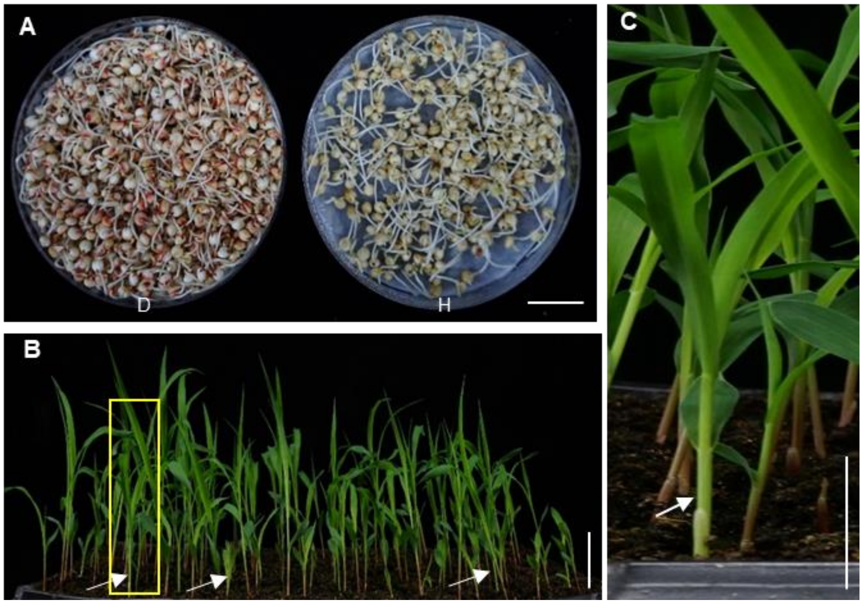

Fig. S23. **Dual haploid identification using GFP and coleoptile pigmentation markers.**
(**A**) GFP screening separated diploid and haploid seeds, followed by visual selection based on coleoptile color: haploids were colorless, diploids purplish-red. (**B** and **C**) Seedlings from L407A×*Sbmatl Sbdmp*; *P89GFP* show green stems in haploids (white arrows) and red stems in diploids. Scale bars: 2 cm (A), 5 cm (B and C).

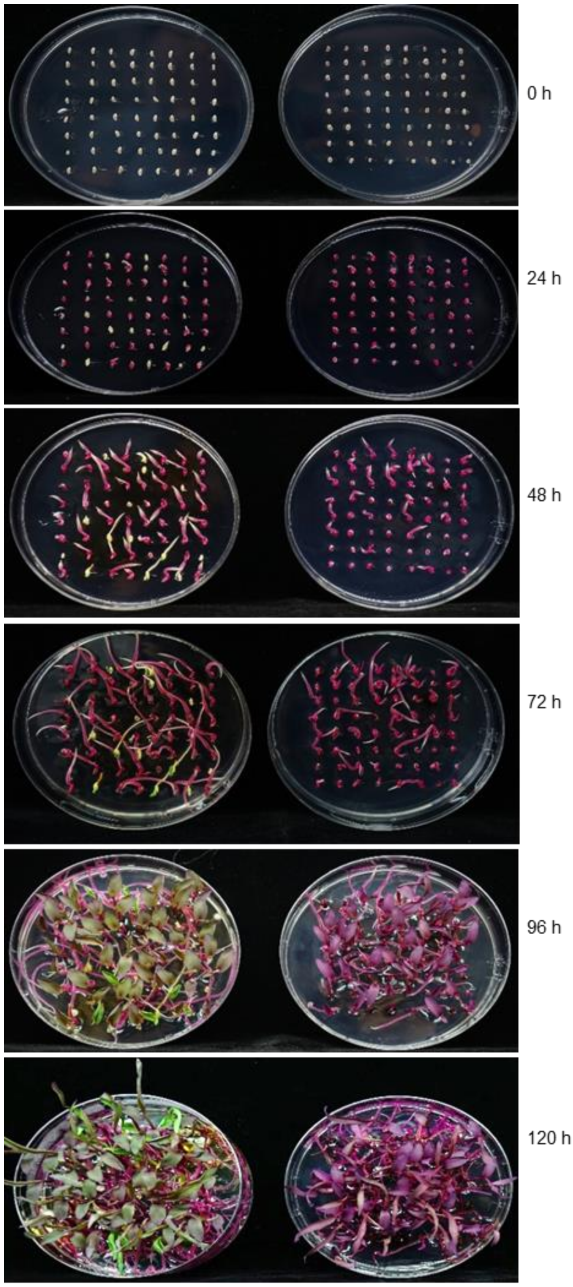

Fig. S24. Haploid embryo identification using RUBY pigmentation during *in vitro* culture. Embryos from L407A × *Sbdmp-1 Sbmatl-2*;*P89GFP-RUBY* (left) and self-pollinated progeny of the same inducer (right) were cultured *in vitro* and imaged at 0, 24, 48, 72, 96, and 120 hours post-culture. The absence of RUBY pigmentation in germinating embryos from pollination (left) allowed visual distinction of haploid from diploid, while all germinating embryos from selfing of the inducer line displayed uniform ruby color (right). Dish diameter, 90 mm.

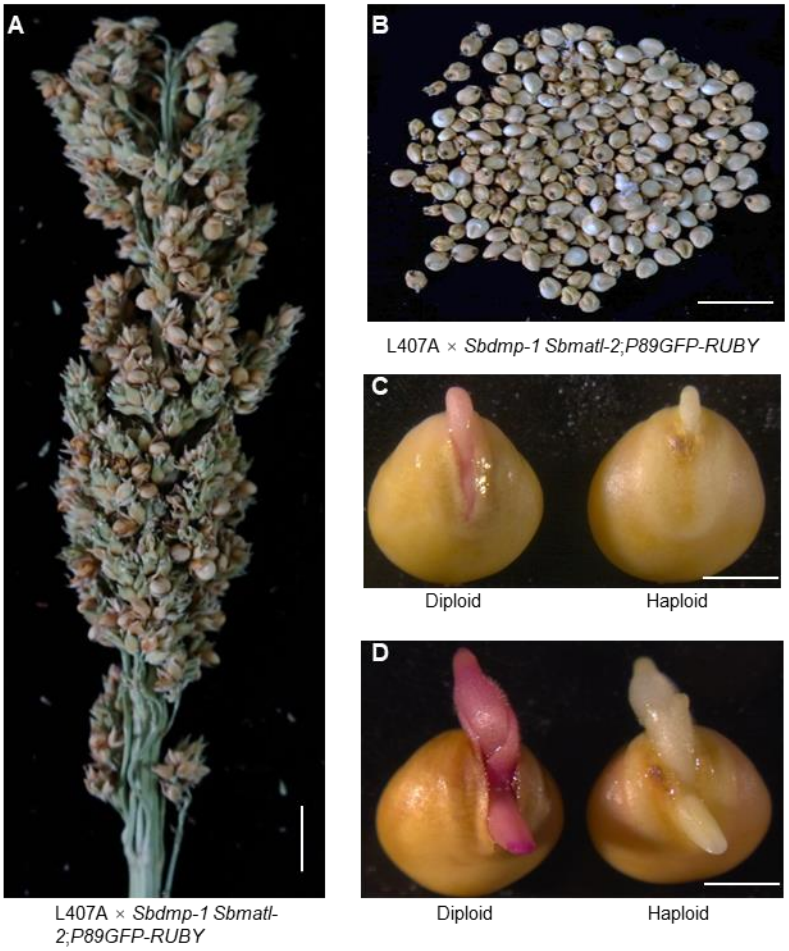

Fig. S25. **Visual identification of haploid seeds using RUBY pigmentation during germination**.

(**A** and **B**) Panicle (A) and mature seeds (B) from L407A × *Sbdmp-1 Sbmatl-2*;*P89GFP-RUBY* cross showing unpigmented maternal seed coats. (**C** and **D**) Germinating seeds at day 1 (C) and day 2 (D): diploid embryos showing strong RUBY pigmentation, while haploids lacking pigmentation. Scale bars: 5 cm (A), 2 cm (B), 2 mm (C and D).

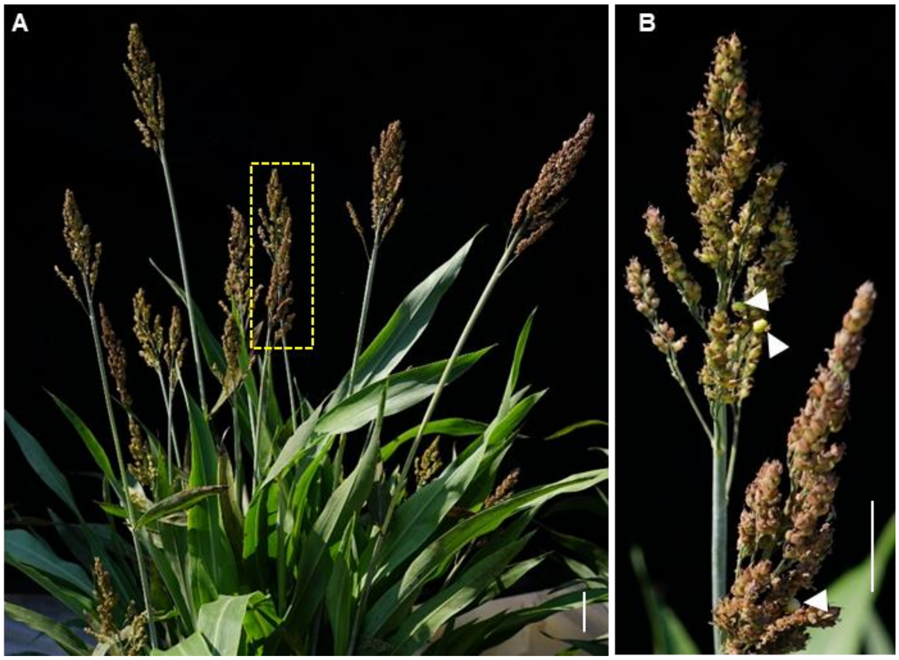

Fig. S26. Ratooning enhances doubled-haploid seed recovery by increasing panicle production.

(**A**) A low-fertility haploid plant transplanted and ratooned in Hainan, showing prolific tillers. (**B**) A close-up view of a panicle in A, showing seed sets white arrowheads by generating new tillers. Scale bars: 20 cm (A), 1 cm (B).

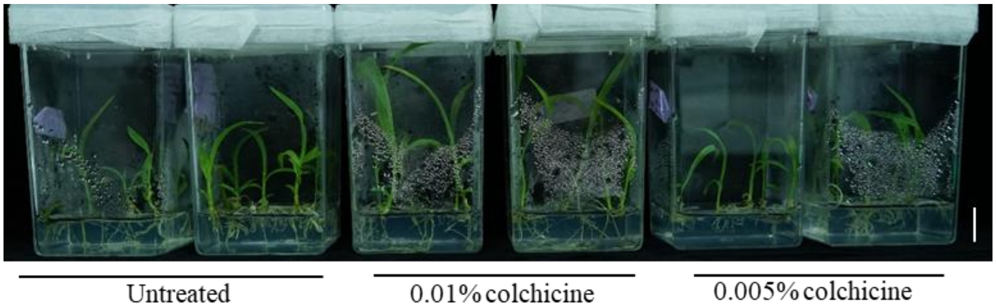

Fig. S27. Germination of haploid embryos treated with colchicine.

Young embryos were isolated from seeds 15 days after pollination with inducer pollens. Haploid embryos were identified using the GFP marker and treated with colchicine for 10 hours. Embryos were germinated on the hormone-free half-strength MS medium for 17 days before photographing. Scale Bar, 2 cm.

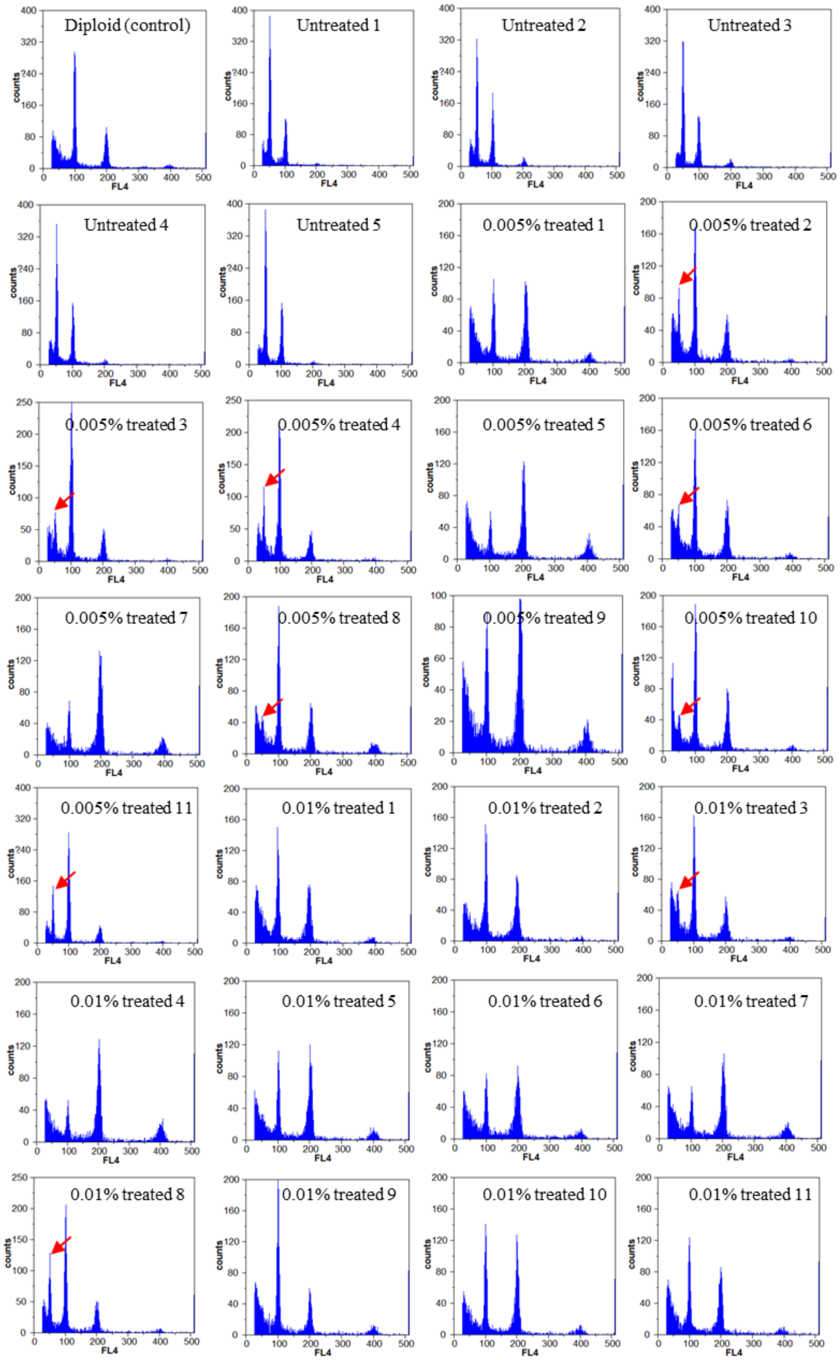

Fig. S28. Plant ploidy level measurement by flow cytometry.

Embryos were germinated for 17 days before flow cytometry analysis. Eleven plants were sampled each for 0.005% and 0.01% colchicine-treated haploid embryos, with plants from one diploid and five untreated haploid embryos as controls. Signal intensities at 50 and 100 represent haploid and diploid, respectively. Chimeric haploid/diploid plants are indicated by red arrows.

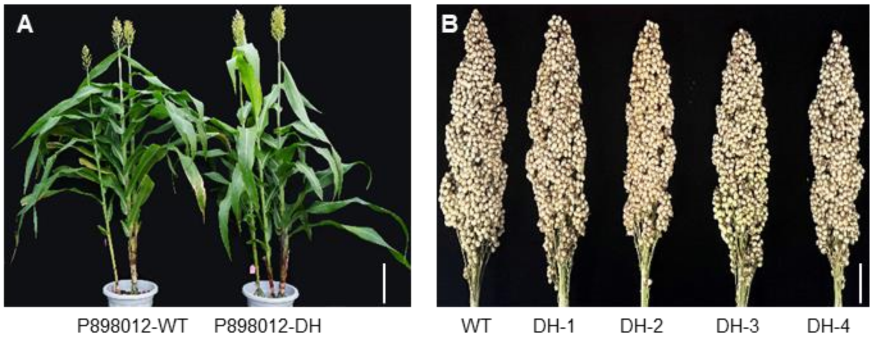

Fig. S29. Production and characterization of DH plants in the P898012 background.
(**A**) DH plant (right) grown from spontaneously doubled seed, compared with wild-type P898012 (left), showing normal vegetative growth. (**B**) Panicles from DH lines display normal morphology and complete seed set, confirming fertility restoration. Scale bars: 10 cm (A), 0.5 cm (B).

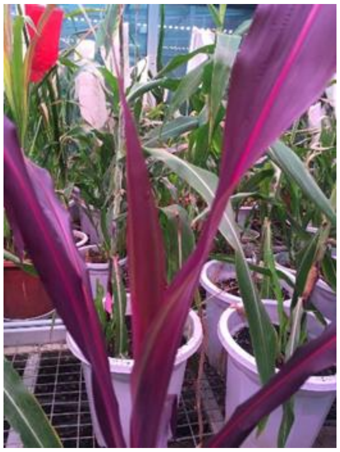

Fig. S30. A RUBY plant stands out from other sorghum plants.

Table S1. (Separated file)

Identification of T-DNA insertion site.

Table S2. (Separated file)

Mutagenesis of *SbMATL* in two sorghum backgrounds.

Table S3 (Separated file)

HIRs of *Sbmatl* mutants in crossing-pollinated progeny.

Table S4. (Separated file)

Generation *Sbdmp*,*Sbmatl Sbdmp* mutant in two sorghum backgrounds.

Table S5. (Separated file)

The details about HIRs in *Sbmatl Sbdmp* of *P89GFP* background.

Table S6. (Separated file)

Generation *Sbpld3, Sbmatl Sbpld3* and *Sbmatl Sbdmp Sbpld3* mutants in *P89GFP* background.

Table S7. (Separated file)

The details of HIR evaluation about SbPLD3 in *P89GFP* background.

Table S8. (Separated file)

Pollen competition assay between inducer and wild-type plants.

Table S9. (Separated file)

Comparison of seed setting rate and aborted rate between wild-type, *Sbmatl, Sbdmp* and *Sbmatl Sbdmp* mutants in *P89GFP* and *TxGFP* background.

Table S10. (Separated file)

Haploid induction rates of *SbMATL* mutants in two sorghum backgrounds at early and mature seed stages.

Table S11. (Separated file)

The details about HIRs of *Sbmatl, Sbdmp* and *Sbmatl Sbdmp* comparison between *P89GFP* and *TxGFP* background.

Table S12. (Separated file)

The details comparison of maternal parents' effection about HIRs in 2022 Beijing summer pot and field trials.

Table S13. (Separated file)

Comparative analysis of environmental effection of HIRs in Beijing and Hainan across different years.

Table S14. (Separated file)

Evaluation of sorghum seed set and self-fertilization following high-humidity emasculation treatment.

Table S15. (Separated file)

High self-fertilization rate following manual emasculation in sorghum.

Table S16. (Separated file)

Evaluation of male sterility induction in sorghum by chemical (TFMSA) treatment.

Table S17. (Separated file)

Efficiency of TFMSA-induced male sterility and cross-pollination success evaluation in sorghum.

Table S18. (Separated file)

Evaluation of repeated treatment on male emasculation.

Table S19. (Separated file)

Chemical emasculation enables wide compatibility in haploid induction across diverse sorghum germplasms.

Table S20. (Separated file)

Female fertility of Spontaneous doubled L407A haploid pollinated with different wild-type pollen sources.

Table S21. (Separated file)

Spontaneous chromosome doubling capability in haploid plants across different genetic backgrounds.

Table S22. (Separated file)

Limited detectable spontaneous seed set in haploid plants across two F_1 breeding hybrids.

Table S23. (Separated file)

Spontaneous chromosome doubling data for 74 haploid sorghum plants across two environments.

Table S24. (Separated file)

Cultivars or varieties used in this study.

Table S25. (Separated file)

Primers used in this study.
